# Tissue-Specific, Development-Dependent Phenolic Compounds Accumulation Profile and Gene Expression Pattern in Tea Plant [*Camellia sinensis*]

**DOI:** 10.1371/journal.pone.0062315

**Published:** 2013-04-30

**Authors:** Xiaolan Jiang, Yajun Liu, Weiwei Li, Lei Zhao, Fei Meng, Yunsheng Wang, Huarong Tan, Hua Yang, Chaoling Wei, Xiaochun Wan, Liping Gao, Tao Xia

**Affiliations:** 1 Key Laboratory of Tea Biochemistry and Biotechnology, Ministry of Education in China, Anhui Agricultural University, Hefei, Anhui, China; 2 School of Life Science, Anhui Agricultural University, Hefei, Anhui, China; 3 Biotechnology Center, Anhui Agricultural University, Hefei, Anhui, China; Wuhan University, China

## Abstract

Phenolic compounds in tea plant [*Camellia sinensis* (L.)] play a crucial role in dominating tea flavor and possess a number of key pharmacological benefits on human health. The present research aimed to study the profile of tissue-specific, development-dependent accumulation pattern of phenolic compounds in tea plant. A total of 50 phenolic compounds were identified qualitatively using liquid chromatography in tandem mass spectrometry technology. Of which 29 phenolic compounds were quantified based on their fragmentation behaviors. Most of the phenolic compounds were higher in the younger leaves than that in the stem and root, whereas the total amount of proanthocyanidins were unexpectedly higher in the root. The expression patterns of 63 structural and regulator genes involved in the shikimic acid, phenylpropanoid, and flavonoid pathways were analyzed by quantitative real-time polymerase chain reaction and cluster analysis. Based on the similarity of their expression patterns, the genes were classified into two main groups: C1 and C2; and the genes in group C1 had high relative expression level in the root or low in the bud and leaves. The expression patterns of genes in C2-2-1 and C2-2-2-1 groups were probably responsible for the development-dependent accumulation of phenolic compounds in the leaves. Enzymatic analysis suggested that the accumulation of catechins was influenced simultaneously by catabolism and anabolism. Further research is recommended to know the expression patterns of various genes and the reason for the variation in contents of different compounds in different growth stages and also in different organs.

## Introduction

Tea is one of the three popular nonalcoholic beverages consumed throughout the world. A great quantity of epidemiological studies indicated that the daily consumption of green tea is considered as part of a lifestyle which supports healthiness and long life [1]–[3]. Research reports suggested that the consumption of tea [*Camellia sinensis* (L.)] had a protective effect on reducing the incidence of all cancers [4], [5], decreasing body weight and body fat [6]–[8], improving glucose homeostasis [9]–[12], and enhancing cardiovascular health [13], [14]. Phenolic compounds are the main flavor components and functional ingredients in tea, which possess key pharmacological activities such as antioxidant, antimutagenic, anticarcinogenic, antidiabetic, antibacterial, anti-inflammatory, antihypertensive, anticardiovascular disease, solar UV protection, body weight control, and therapeutic properties in Parkinson’s disease [15].

According to the differences in chemical structure, flavonoids can be differentiated into several subgroups including chalcones, flavones, flavonols, flavanols, anthocyanins, condensed tannins or proanthocyanidins (PAs), and other specialized forms of flavonoids [16]. Phenolic compounds, an important secondary metabolite in tea, account for 18% to 36% dry weight in the fresh leaves and tender stem. More than 96 phenolic compounds have been identified from 41 green teas and 25 fermented teas, which included the following: 28 acylated glycosylated flavonols, 19 O-glycosylated flavonols, 15 phenolic acid derivatives, 12 catechins, 7 C-glycosylated flavones, 6 PAs, and 3 flavonols [17]. In addition, the methylated catechins have also been identified in fresh leaves and other tea products [18].

**Figure 1:**
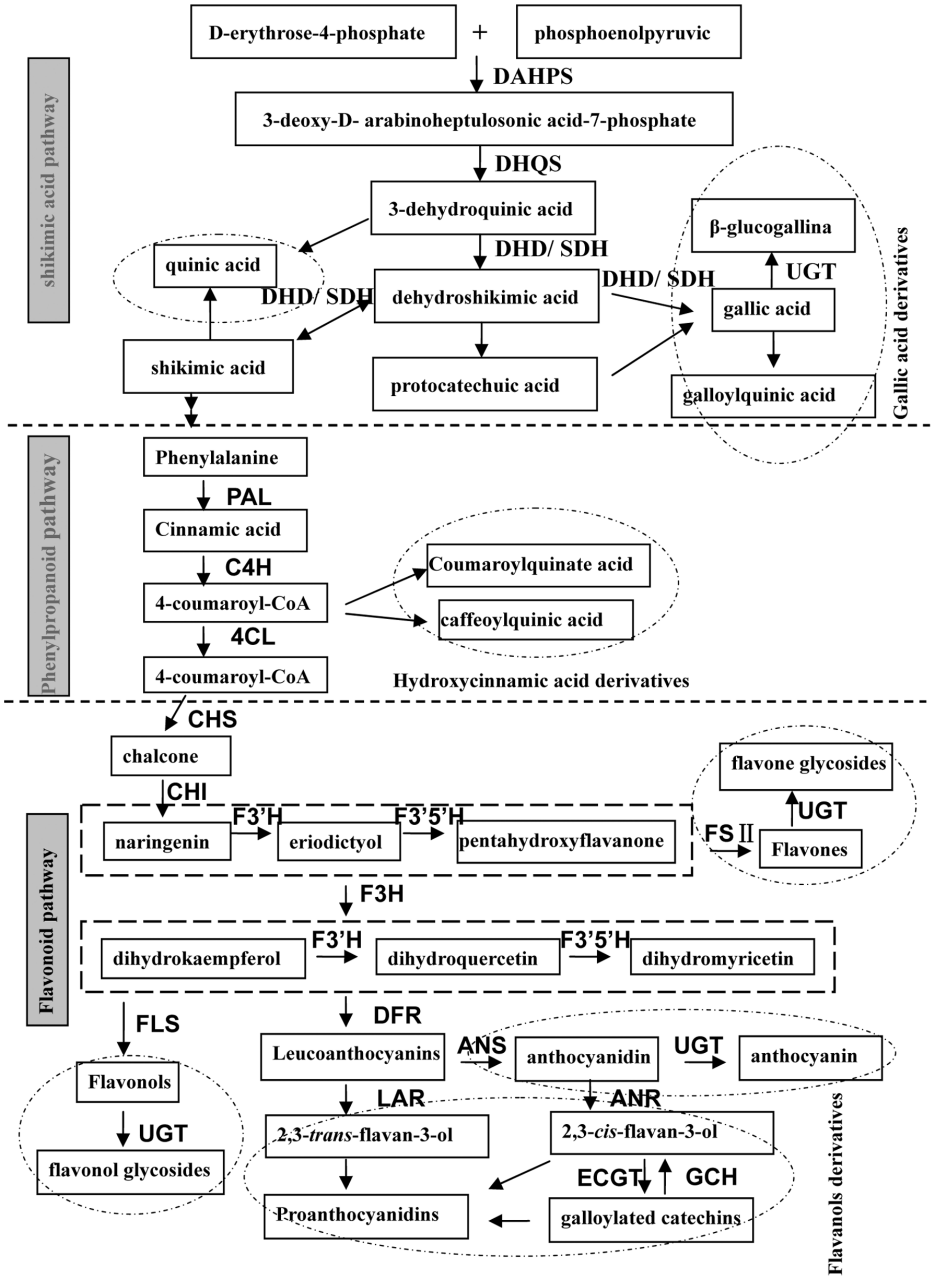
Biosynthesis pathway of phenolic compounds in tea plant. Notes: Enzymes: DAHPS, 3-deoxy-D-arabinoheptulosonate 7-phosphate synthase; DHQS, 3-dehydroquinate synthase; DHD, 3-dehydroquinate dehydratase; SDH, shikimate dehydrogenase; PAL, phenylalanine ammonia lyase; C4H, cinnamate 4-hydroxylase; 4CL,4-coumaroyl-CoA ligase; CHS, chalcone synthase; CHI, chalcone isomerase; F3H, flavanone 3-hydroxylase; F3’H, flavonoid 3′-hydroxylase; F3’5’H, flavonoid 3′,5′-hydroxylase; DFR, dihydroflavonol 4-reductase; LAR, leucoanthocyanidin reductase; ANS, anthocyanidin synthase; ANR, anthocyanidin reductase; UGT, uridine diphosphate Glycosyltransferase; GCH, galloylated catechins hydrolase; ECGT, epicatechins 1-*O*-galloyl-β-D-glucose *O*-galloyltransferase.

All phenolic compounds share similar biosynthetic pathways such as shikimic acid pathway, phenylpropanoid pathway and flavonoid synthetic pathway (in order) (**Fig. 1**). Phenolic compounds, arising from different branch paths are regulated by structural genes, multifunctional transcription factor and regulator protein. Since the late 20th century, biosynthesis pathway of phenolic compounds have been an efficient model for studying various biological processes [19], metabolic pathways [16] and regulatory networks [20].

Extensive studies on model organisms like tobacco, *Arabidopsis thaliana*, and *Medicago truncatula* facilitated the understanding of regulation system and subcellular location in the flavonoid pathway [21]–[23]. In the recent years, more number of researches were conducted on the synthetic regulation, transport and galloylation of phenolic compounds, and polymerization of PAs synthesis [6], [23]–[28]. There are similarities and differences in biosynthetic pathway of phenolic compounds between model plants and tea plant. For instance, the galloylated catechins, including epigallocatechin gallate (EGCG) and epicatechin gallate (ECG), account for up to 76% of catechins in the tea plant [29], [30]. It has been believed that the flavonoids are synthesized exclusively in the endoplasmic reticulum followed by the transfer to vacuoles for bio-processes by multidrug resistance-associated protein or multidrug and toxic compound extrusion proteins [31], [32]. However, catechins were possibly located mainly in the chloroplasts of mesophyll cells and vessel walls rather than the vacuoles [33]. Light radiation had no significant effect on the accumulation of anthocyanin and galloylated catechins in tea plant grown in outside field [34], which was inconsistent from the other model organisms.

Little research has been specialized in the function of structural genes and regulatory genes in the metabolism of phenolic compounds in tea plant, and a very few structural and regulatory genes have been logged in the National Center of Biotechnology Information (NCBI) database. A *C.sinensis* transcriptome database containing 127,094 unigenes has been obtained recently by high-throughput Illumina for large-scale ribonucleic acid (RNA) sequencing in the Key Laboratory of Tea Biochemistry and Biotechnology, and a large number of predicted structural and regulatory genes related to the metabolism of phenolic compounds have been screened out from this database [35]. A genome-wide bioinformatic analysis of transcription factors in the biosynthesis of phenolic compounds has been published [36].

In addition, a recently published article suggested that the galloylated catechins were biosynthesized in the tea plant via 1-*O*-glucose ester-dependent two-step acyltransferase reactions, which involved two newly discovered enzyme, UDP-glucose: galloyl-1-*O*-β-D-glucosyltransferase (UGGT) and epicatechin: 1-*O*-galloyl-β-D-glucose *O*-galloyltransferase (ECGT) [37]. High activity of galloylated catechins hydrolase (GCH) was detected in tea plant by *in vitro* enzymatic activity analysis [38].

In order to predict function of these genes, the profile of phenolic compounds in tea plant were established by employing liquid chromatography in tandem mass spectrometry technology, and the expression pattern of potential genes were investigated using quantitative real-time polymerase chain reaction (qRT-PCR) techniques and classified by clustering analysis. In addition, the metabolism of galloylated catechins was researched by *in vitro* enzymology analysis. The present research attempted to merge genetic and metabolic analyses and get insight into biological connections between genetic and biochemical pathways.

## Materials and Methods

### Plant Materials

Based on the extent of growth maturity of *C. sinensis*, the following samples were collected from more than 3 year old tea plants during early summer from the Experimental Tea Garden, Anhui Agricultural University, Anhui, China (latitude: 31.86N, longitude: 117.27E, altitude: 20 m above mean sea level): young shoots (bud, first leaf, second leaf, third leaf, fourth leaf, young stems), and tender roots. The collected samples were immediately frozen in liquid nitrogen and stored at −80°C prior to analysis.

### Chemicals and Reagents

Catechin, epicatechin, gallocatechin, epigallocatechin, epigallocatechin gallate, epicatechin gallate, quinic acid, β-glucogallin, galloylquinic acid, caffeoylquinic acid, galloyl acid, caffeine, quercitrin, myricetrin and kaempferitrin were obtained from Sigma (St Louis, MO, USA). Cyanidin chloride and delphinidin chloride were obtained from Axxora Co., Ltd (Lausanne, Switzerland). HPLC grade methanol, acetonitrile and acetic acid were obtained from Tedia Co., Ltd (Fairfield, OH, USA).

### Extraction and Identification of Phenolic Compounds

The total phenolic compounds were extracted as follows: 0.5 g sample (fresh leaves, young stem, and root) was grounded in liquid nitrogen and extracted with 5 mL extraction solution [80% methanol:1% hydrochloric acid (HCl)] using an ultrasonic sonicator for 10 min at room temperature. After centrifugation at 4000 *g* for 15 min, the residues were re-extracted twice as above, and the supernatants were filtered through a 0.22 µm membrane.

The liquid chromatography (LC)-time of flight (TOF)-mass spectrometer (MS) system used in this study consisted of a quaternary pump with a vacuum degasser, thermostated column compartment, autosampler, diode array detector (DAD), and TOF-MS from Agilent Technologies (Palo Alto, CA, USA). A Phenomenex Synergi 4 u Fusion-RP80 column (particle size: 5 µm, length: 250 mm, and internal diameter: 4.6 mm) was used at a flow rate of 1.0 mL min^−1^. The column oven temperature was set at 25°C. The mobile phase consisted of 1% acetic acid in water and 100% acetonitrile; and the gradient of the latter increased linearly from 0 to 10% (v/v) at 10 min, to 13% at 30 min, to 16% at 65 min, to 33% at 81 min, to 90% at 85 min, and to 90% at 90 min. The DAD was set at 280 nm and 340 nm for real-time monitoring of the peak intensities. Ultraviolet (UV) spectra were continuously recorded from 200 nm to 600 nm for the identification of plant components. Mass spectra were acquired simultaneously using the electrospray ionization in the positive and negative ionization modes at the fragmentation voltages of 175 V over the range of *m/z* 100 to 2000. A drying gas flow of 12 L min^−1^, drying gas temperature of 325°C, nebulizer pressure of 35 psi, and capillary voltages of 3500 V were used.

The ultra performance liquid chromatography (UPLC)-MS/MS system used in this study consisted of a quaternary pump with a vacuum degasser, thermostated column compartment, autosampler, DAD, and triple quadrupole Mass Spectrometer (QQQ) from Agilent Technologies (Palo Alto, CA, USA). An Agilent 20RBAX RRHD Eclipse Plus C18 column (particle size: 1.8 µm, length: 100 mm, and internal diameter: 2.1 mm) was used at a flow rate of 0.2 mL min^−1^. The column oven temperature was set at 40°C. The mobile phase consisted of 0.4% acetic acid in water and 100% acetonitrile; and the gradient of latter increased linearly from 0 to 7% (v/v) at 10 min, and to 7% at 22 min, to 11% at 25 min, to 12% at 30 min, to 14% at 31 min, to 35% at 43 min, and to 80% at 47 min. Mass spectra were acquired simultaneously using electrospray ionization in the positive and negative ionization modes over the range of *m/z* 100 to 2000. A drying gas flow of 6 L min^−1^, drying gas temperature of 350°C, nebulizer pressure of 45 psi, and capillary voltages of 3500 V were used.

The phenolic compounds were identified qualitatively using LC-MS by comparing the retention times (t_R_), wavelengths of maximum absorbance (λmax), protonated/deprotonated molecules ([M+H]^+^/[M–H]^−^), and major fragment ions with those of the authentic standards and published literature.

### Extraction and Quantitative Determination of Phenolic Compounds

MS-based multiple reaction monitoring (MRM) mode was used for simultaneous quantitation of phenolic compounds. The extraction method and MS-MRM conditions were similar to the above described qualitative analysis.

Spectrophotometry analysis of anthocyanins was performed as suggested by Pang et al. [24]. Total anthocyanin concentration was calculated using the molar absorbance of cyanidin-3-*O*-glucoside (XinRan Biological, Shanghai, China).

The PAs were extracted as reported by the methods of Martin et al. [39] and Pang et al. [24] with some modifications. The sample (0.5 g each of fresh leaves, stems, and roots) was grounded in liquid nitrogen and extracted with 5 mL extraction solution (70% acetone:0.5% acetic acid) by sonication for 10 min at room temperature. The extract was centrifuged to remove the debris, and the residues were re-extracted twice as mentioned above.

For the analysis of soluble PAs, 1 mL of pooled supernatant added with 1 mL of water was extracted with 2 mL of chloroform. Aqueous supernatant (0.5 mL) was then added to 3 mL *n*-butanol-HCl (95/5 v/v) and incubated at 95°C for 1 h. The supernatant was cooled to room temperature, and the absorbance at 550 nm and 600 nm were recorded. The control treatment was performed under same conditions without boiling. Absorbance values were converted into PAs equivalents using a standard curve of procyanidin B2.

For the analysis of insoluble PAs, the residues were added to 3 mL *n*-butanol-HCl (95/5 v/v) and incubated at 95°C for 1 h. The detection method used was similar to that of soluble PAs. The hydrolysates were then subjected to LC-MS analysis (as described above).

### Expression difference of Genes Involved in Phenolic Compounds Biosynthesis by qRT-PCR

Total RNA was isolated from *C. sinensis* with RNAiso-mate for Plant Tissue (Takara, DaLian, China; Code: D325A) and RNAiso Plus (Takara, DaLian, China; Code: D9108B). Complementary deoxyribonucleic acid (cDNA) strands for qRT-PCR were synthesized with PrimeScript® RT reagent Kit (Takara, DaLian, China; Code: DRR037A).

The primer sequences are listed in **Table S1**. The PCR mixture contained cDNA template (approximate 0.01 µg/µL), 10 µL SYBR Green PCR Master Mix (Takara, DaLian, China), and 200 nmol L^–1^ of each gene-specific primer in a final volume of 20 µL. qRT-PCR assays were performed using a CFX96™ optical reaction module (Bio-RAD, USA). The PCRs were performed with the following program: 95°C for 30 sec, followed by 40 cycles at 95°C for 5 sec, and 60°C for 30 sec (58°C for 30 sec for root) in 96-well optical reaction plates. The specificity of amplicons was verified by melting curve analysis (55°C to 95°C). Expression of messenger RNA was assessed by evaluating threshold cycle (C_T_) values in quadruplicate reactions. Values were normalized against the expression level of the housekeeping gene glyceraldehyde-3-phosphate dehydrogenase (*GAPDH*). The relative expression values were evaluated by the 2^−△△Ct^ method: △C_T_ = C_ T, target_ -C_T, GAPDH_, -△△C_T_  = -(△C_T, target_ -△C_T, bud_), where C_ T, target_ and C_T, GAPDH_ were the threshold cycles of targets and housekeeping gene *GAPDH*, respectively.

The experiments were repeated thrice.

### Clustering Method

The hierarchical cluster of the gene expression data was performed using the between-groups linkage method of Statistical Package for the Social Sciences statistics17.0.

### Enzymatic Assays of UGGT, ECGT and GCH

Enzyme extraction was performed in accordance with the method of Liu et al and Zhang et al [37], [40]. All enzyme assays were conducted in phosphate buffer.

The UGGT and ECGT reactions solution were prepared as mentioned by Liu et al [37]. The GCH assay solution was incubated at 30°C for 0.5 h in a total volume of 2.5 mL containing 50 mM phosphate buffer (pH 6.5), 0.2 mM EGCG or ECG, 4 mM ascorbic acid, and crude enzyme extract (0.4 mg total protein).

The above enzyme reactions were terminated by adding ethyl acetate. Each of the reaction products was extracted thrice with 3 mL ethyl acetate, and the extract was evaporated and redissolved in 500 µL methanol and used directly for the analyses of enzymatic reaction products.

The experiments were repeated thrice.

## Results

### Identification of Phenolic Compounds via LC-TOF-MS and UPLC-QQQ-MS/MS

**Figure 2:**
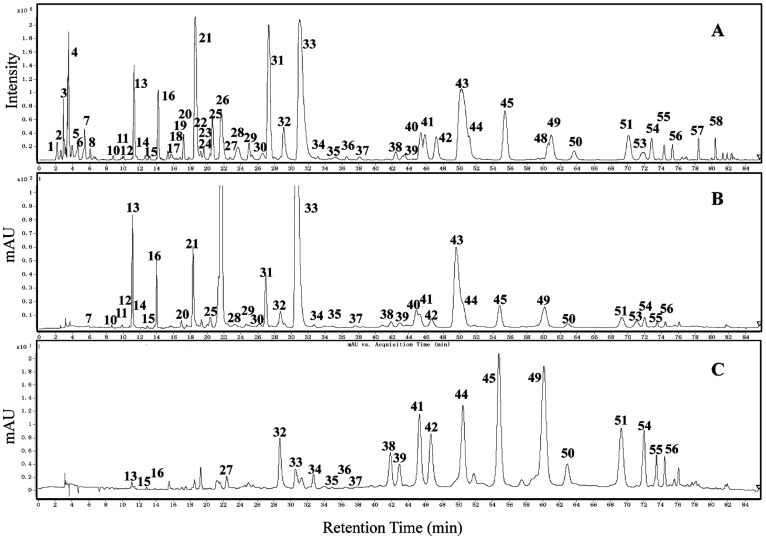
Chromatogram of HPLC-TOF-MS for phenolic compounds in tea leaves: (A) Total ion chromatography, (B) HPLC Chromatogram at 280 nm, and (C) HPLC Chromatogram at 340 nm. The sample preparation methods are described in the Materials and Methods. Compounds with peaks are listed in Table 1.

**Figure 3:**
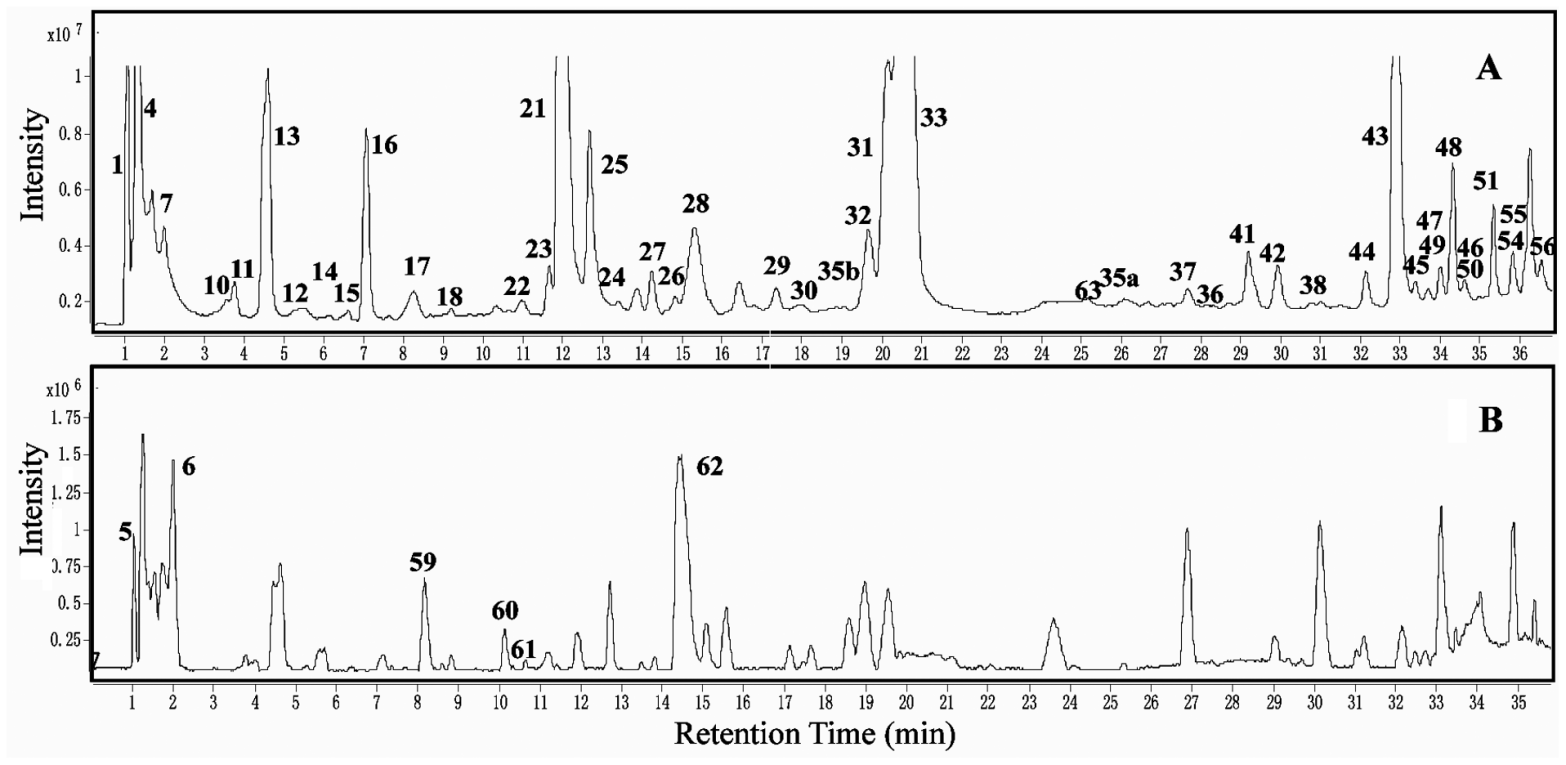
Chromatogram of UPLC-QQQ-MS/MS for phenolic compounds in tea leaves: (A) Total ion chromatography in negative mode and (B) Total ion chromatography in positive mode. The sample preparation methods are described in the Materials and Methods. Compounds with peaks are listed in Table 1.

Phenolic compounds in tea plant were analyzed qualitatively by LC-TOF-MS and UPLC-QQQ-MS/MS, and they were identified (**Table 1**, **Figs. 2** and **3**) based on the data obtained from the standard substances or published literature including t_R_, λmax, ([M+H]^+^/[M–H]^−^), and major fragment ions [17], [41]–[44].

**Table 1:** Analyses of phenolic compounds in tea leaves (shown in Figures 2 and 3).

Peak/no.	t_R_^1^ (min)	t_R_^2^ (min)	[M–H]^+^/[M–H]^−^ (*m/z*)	MS/MS (*m/z*)	UV λmax (nm)	identification
1	2.182	1.100	-/343.05211	-/191	276	galloylquinic acid
2	2.587	-	-/156.99868	-/-	220	unkuown
3	2.925	-	-/311.09482	-/151,169,270	225	unkuown
4	3.532	1.280	-/191.05294	-/85,127	-	quinic acid
5	3.954	1.037	-/173.08845	111,130,158/-	-	theanine^a^
6	4.629	2.010	-/173.09049	111,130,158/-	-	theanine^a^
7	5.440	1.608	-/191.01286	-/85,127	-	quinic acid^a^
8	6.098	-	-/203.01703	-/-	270	unkuown
9	6.203	-	-/128.03493	-/-	-	unkuown
10	8.815	3.778	-/331.06123	-/151,169, 270	276	β-glucogallin^a^
11	10.098	-	-/169.01302	-/125	274	galloyl acid^a^
12	11.060	5.164	-/609.11332	-/125,305,423	272	EGC-EGC
13	11.313	4.508	-/343.08292	-/191	276	galloylquinic acid^a^
14	12.613	6.408	-/467.11105	-/243,305	220	gallocatechin-glucoside
15	13.203	6.424	-/609.11591	-/169,305,331,457	275	EGCDG
16	14.166	7.048	-/305.06385	-/125, 165,179,219	275	gallocatechin^a^
17	15.735	8.054	-/609.11583	-/169,305,331,457	275	EGCDG
18	16.292	10.382	-/593.15606	-/289,305, 407,423	270	EC-EGC
19	16.765	12.079	-/425.03299	-/169,273	274	epiafzelechin gallate
20	17.187	-	-/357.07750	-/-	275	Unkuown
21	18.587	11.356	-/305.06517	-/125,137,179, 219	275	epigallocatechin^a^
22	19.229	11.075	-/593.12065	-/169,289,441	270	ECDG
23	19.600	11.102	-/337.10788	-/163,173, 191	310	p-coumaroylquinic acid
24	20.343	11.903	-/761.12183	-/125,305,423,591	275	EGC-EGCG
25	20.630	12.044	-/289.06836	-/109,125,203,245	278	catechin^a^
26	21.659	13.388	-/316.04905	-/	275	Unkuown
27	22.739	13.208	-/353.08317	-/191	240,326	caffeoylquinic acid^a^
28	23.668	13.896	-/577.12389	-/125,289,407,425,451	273	EC-EC dimmer
29	25.018	15.272	-/577.12284	-/125,289,407,425,451	273	PAs B2 a
30	26.621	15.631	-/745.12819	-/169,407,423,593	278	ECG-EGC
31	27.330	17.409	-/289.07148	-/109,125,203,245	278	epicatechin^a^
32	29.086	16.351	-/337.08760	-/163,173, 191	310	p-coumaroylquinic acid
33	30.993	17.695	-/457.09664	-/125,169,305	278	epigallocatechin gallate^a^
34	33.153	33.153	-/593.14276	-/455,473	272,336	6,8-*C*-diglucosylapigenin
35a	34.908	16.213	-/865.18022	-/125,407	278	EC-EC-EC
35b	35.280	22.881	-/729.13377	-/125,245,407,577	278	EC-ECG
36	36.596	28.435	-/273.07432	-/97,229	275	Epiafzelechin
37	38.132	25.199	-/729.17308	-/289,407, 441,559	278	EC-ECG
38	42.352	-	-/563.16401	-/353,443	272,336	apigenin 6-*C* glucosyl-8-*C*-arabinoside
39	43.499	31.628	-/563.13169	-/353,443	272,336	apigenin 6-*C*-arabinosyl-8-*C*-glucoside
40	45.390	-	-/471.11295	-/125,169, 287, 305	270,350	Unkuown
41	45.896	29.165	-/479.10240	-/317	262,356	myricetin 3-*O*-galactoside
42	47.246	29.951	-/479.07437	-/317	262,356	myricetin 3-*O*- glucoside
43	50.200	31.464	-/441.10108	-/125,169, 271,289	278	epicatechin gallate^a^
44	51.145	32.150	-/771.22626	-/301	256,354	quercetin 3-*O*-galactosylrutinoside
45	55.415	33.402	-/771.22746	-/301	256,354	quercetin 3-*O*-glucosylrutinoside
46	59.854	34.550	-/577.17831	-/311,431	270,338	vitexin-2"-*O*-rhamnoside
47	60.850	34.226	-/609.17014	-/301	256,354	quercetin-3-*O*-rutinoside
48	60.546	34.341	-/755.23224	-/285	266,348	kaempferol 3-*O*-galactosylrutinoside
49	60.917	34.006	-/463.10548	-/301	258,354	quercetin 3-galactoside
50	63.702	34.602	-/463.08048	-/301	258,354	quercetin 3-*O*-glucoside
51	70.099	35.362	-/755.23010	-/285	266,348	kaempferol 3-*O*- glucosylrutinoside
52	72.668	-	-/600.37987	-/-	-	unkuown
53	71.888	-	-/455.11536	-/-	278	methoxyepiafzelechine gallate
54	72.850	35.831	-/447.08760	-/285	266,348	kaempferol-3-*O*-galactoside
55	74.318	36.122	-/593.14242	-/285	266,348	kaempferol 3-*O*-rhamnosylgalactoside
56	75.314	36.562	-/447.08732	-/285	266,348	kaempferol-3-*O*-glucoside
57	78.403	-	-/737.50451	-/-	220	unkuown
58	80.428	-	-/850.58625	-/-	220	unkuown
59	15.923	8.202	181.00886/-	113,138/-	274	theobromine or theophylline
60	16.102	10.086	611.14039/-	303/-	524	delphinidin 3-rutinoside
61	16.546	10.636	595.14418/-	287/-	524	cyanidin 3-rutinoside
62	24,568	14.478	195.08870/-	138/-	268	caffeine^a^
63	35.823	27.522	-/897.15219	-/-	278	ECG-EGCG or EGC-EGC-EC

a Compounds positively identified by direct comparison with the standard; other compounds were approximately identified qualitatively by comparing the wavelengths of maximum absorbance, protonated and deprotonated molecules ([M–H]^+^/[M–H]^−^), and fragment ions (PI/NI) from the published literature; the underlined daughter ion was the ion for quantitative determination; t_R_^1^ (min) was the retention time in **Figure 2**; and t_R_^2^ (min) was the retention time in **Figure 3**. [M–H]^+^/[M–H]^−^ (*m/z*) of compounds were detected by LC-TOF-MS; MS/MS (*m/z*) of compounds were detected by UPLC-TOF-MS/MS.

A total of 64 metabolites were detected successfully by one-time measurement. Of which, 50 were identified as phenolic compounds (**Table 1**). All the samples were analyzed in negative ionization mode except for anthocyanidin, theanine, caffeine, theobromine, and theophylline, which were obtained in positive ionization mode.

Nine phenolic acids among the 50 phenolic compounds consisted of hydroxybenzoic acid (HBA) and hydroxycinnamic acid (HCA) derivatives, and they were identified by direct comparison of data with authentic standards (**Table 1**, **Figs. 2** and **3** **, Fig. S1**) and literature [17]. The gallic acid (GA) derivatives which were belonging to HBA derivatives included the following: GA (peak 11), β-glucogallin (βG, peak 10), and galloylquinic acid (GQA, peaks 1 and 13). HCA derivatives comprised of p-coumaroylquinic acid (peaks 23 and 32) and caffeoylquinic acid (peak 27). Quinic acid (peaks 4 and 7) had no effectual absorption spectra at the UV-Visible spectroscopy, but could be detected with the MS.

The identity of eight monomers of flavan-3-ol (catechins) was confirmed by direct comparison of their absorbance spectra and t_R_ with the authentic standards (**Table 1**, **Figs. 2** and **3** **, Fig. S2**). The main types were di- or tri- hydroxyl flavan-3-ol in the B-ring, which included EGCG (peak 33), ECG (peak 43), EC (peak 31), EGC (peak 21), C (peak 25), and GC (peak 16). Epiafzelechin gallate (peak 19) and epiafzelechin (peak 36) were low mono-hydroxyl content in the B-ring. Among them, EGCG were the major characteristic phenolic compounds in tea plant which were also the functional ingredient of tea beverages.

Peaks 15, 17 and 22 were predicted as flavan-3-ol di-galliate. Although [M–H]^−^ at *m/z* 609 had been conjectured as gallocatechin dimer by Lin [17], the peaks 15 and 17 were more likely to be tri-hydroxyl flavan-3-ol di-galliate on the basis of [M–H]^−^ at *m/z* 609 and fragment ions at *m/z* 169, 305 and 457 [45], [46]. Peak 22 was probably di-hydroxyl flavan-3-ol di-galliate according to [M–H]^−^ at *m/z* 593 and fragment ions at *m/z* 169, 289 and 441 [46].

A few methylated catechins and glycosylated catechins were detected. According to the absorbance spectra, t_R_ and mass spectra ([M–H]^−^ at *m/z* 467 and 455), peaks 14 and 53 were speculated as the gallocatechin-glucoside and methoxyepiafzelechine gallate, respectively.

A total of 10 predicted oligomeric PAs were detected (**Table 1** **and Fig. S3**). Both TOF-MS and QQQ-MS/MS could only detect oligomeric PAs (**Figs. 2** and **3**), which were rich in component but poor in content in the leaf. Among them, peaks 28 and 29 were identified as catechin drimer by comparison of [M–H]^−^ at *m/z* 577 and fragment ions at *m/z* 289, 425, 407, and 451 [17]. For the same reason, peaks 18 and 22 (*m/z* 593), 35a and 37 (*m/z* 729), 30 (*m/z* 745), 24 (*m/z* 761), 35b (*m/z* 865), and 63 (*m/z* 897) were also speculated as PAs [17], [47], [48], respectively.

About 12 flavonol derivatives with mono- to tri- hydroxyl in the B-ring were detected in the fresh tea leaves (**Table 1** **and Fig. S4**). Flavonol derivatives, which were abundant in components, mainly existed in the shape of flavonoids such as kaempferol glycosides (peaks 46, 48, 51, 54, 55, and 56), quercetin glycosides (peaks 44, 45, 47, 49, and 50), and myricetin glycosides (peaks 41 and 42) [17], [42].

Besides, 4 flavone glycosides (peaks 38, 39, and 46) and 2 anthocyanins (peaks 60 and 61) were detected [17], [49] in low content (**Table 1**, **Figs. 2** and **3**).

#### Quantitative Determination of Phenolic Compounds via UPLC-QQQ-MS/MS

To gain insight into the biological relationship between the metabolism of phenolic compounds and gene expressions, the accumulation of phenolic compounds were comprehensively measured in the different developmental stages of leaves and different organs (Table 2).

**Table 2:** Content of phenolic compounds in different organs and leaves at different developmental stages.

Compound	bud	1st leaf	2nd leaf	3rd leaf	4th leaf	stem	root
**Phenolic acid (mg/g)**							
Quinic acid (peak 4)	3.84±0.11	4.39±0.32	8.75±0.79	6.59±0.75	7.44±0.53	11.42±0.87	ND
Gallic acid derivatives							
β-glucogallin (peak 10)	2.70±0.09	0.41±0.05	0.31±0.02	0.21±0.01	0.28±0.01	0.04±0.01	ND
galloyl acid (peak 11)	0.11±0.01	0.06±0.01	0.03±0.00	0.02±0.00	0.02±0.00	0.01±0.00	ND
galloylquinic acid (peak 13)	41.39±0.55	31.69±1.02	8.1±0.56	6.12±0.50	4.84±0.38	4.81±0.40	0.08
Summation	44.2±0.65	32.16±1.08	8.44±0.58	6.36±0.51	5.14±0.39	4.83±0.41	0.08
Hydroxycinnamic acids derivatives							
caffeoylquinic acid (peak 27)	0.03±0.00	0.06±0.01	0.02±0.00	0.02±0.00	0.01±0.00	0.01±0.00	ND
p-coumaroylquinic acid (peak 32)	0.86±0.06	1.01±0.08	0.37±0.02	0.58±0.03	0.17±0.01	0.08±0.01	ND
Summation	0.89±0.06	1.07±0.09	0.39±0.02	0.60±0.03	0.18±0.01	0.09±0.01	ND
**Flavanols (mg/g)**							
Nongalloylated Catechins							
catechin (peak 25)	1.08±0.09	0.66±0.05	0.27±0.02	0.17±0.05	0.05±0.00	0.87±0.01	ND
gallocatechin (peak 16)	2.96±0.15	4.23±0.32	3.95±0.05	3.11±0.03	2.96±0.09	1.91±0.02	ND
epicatechin (peak 31)	5.24±0.56	5.04±0.22	6.90±0.05	6.67±0.06	5.48±0.04	5.93±0.05	2.44±0.03
epigallocatechin (peak 21)	11.51±0.65	13.42±0.91	15.81±0.12	15.41±0.02	14.26±0.10	10.08±0.08	ND
Summation	20.79±1.45	23.34±1.50	26.93±0.24	25.35±0.16	22.76±0.23	18.79±0.16	2.44±0.03
Galloylated catechins							
epicatechin gallate (peak 43)	20.65±0.69	17.51±1.59	12.70±0.46	12.74±0.36	11.10±0.75	4.17±0.18	0.08±0.01
epigallocatechin gallate (peak 33)	84.28±2.78	85.82±2.18	73.72±2.65	69.86±0.28	68.10±2.36	23.26±0.29	0.34±0.02
Summation	104.94±3.47	103.33±3.77	86.42±3.11	82.60±0.64	79.20±3.11	27.43±0.47	0.42±0.03
total Catechins	125.73±4.92	126.67±5.27	113.35±3.35	107.95±0.80	101.96±3.34	46.22±0.63	2.86±0.06
**Proanthocyanidins (area)**							
* m/z* 865 (peak 35b)	555±25	393±56	319±53	214±13	145±34	546±67	2404±275
* m/z* 577 (peak 28)	57272±345	41092±579	32018±278	28614±76	19409±245	52289±345	6565±99
* m/z* 729 (peak 37)	7206±21	3537±145	1595±236	1513±59	1129±87	2598±102	ND
* m/z* 593 (peak 18)	5901±35	5188±178	7173±97	4805±601	3761±512	6068±89	ND
PAs B2 (peak 29)	9689±245	7522±78	6729±67	6034±57	4034±86	10699±106	45630±526
* m/z* 761 (peak 24)	2629±56	3432±35	2484±75	1952±98	1825±90	811±98	ND
* m/z* 745 (peak 30)	921±24	1012±98	544±56	456±52	364±89	192±56	ND
PAs (Anthocyanosides reaction) mg/g	14.57±0.03	14.11±0.05	11.00±0.02	9.83±0.01	8.38±0.02	28.35±0.35	47.28±1.23
**flavonols derivatives (area)**							
tri-hydroxyl in B-ring							
myricetin 3-*O*-galactoside (peak 41)	3760±145	11221±435	11495±218	9467±105	9572±187	2104±96	ND
myricetin 3-*O*- glucoside (peak 42)	2197±102	12154±256	20930±109	21254±267	21210±97	1177±108	ND
Summation	5957±247	23375±691	32425±327	30721±372	30782±284	3281±204	ND
di-hydroxyl in B-ring							
quercetin3-*O*-galactosylrutinoside (peak 44)	870±45	1996±78	5024±98	5405±106	3198±167	573±34	50±5
quercetin 3-*O*-glucosylrutinoside (peak 45)	1896±49	6954±145	10758±523	11287±104	7489±88	741±45	36±2
quercetin 3-galactoside (peak 49)	1582±156	6007±201	9083±99	7407±78	7126±97	1549±86	64±5
quercetin 3-*O*-glucoside (peak 50)	1527±89	8868±167	16652±105	16073±167	12826±213	995±93	241±12
Summation	5875±339	23825±591	41517±825	40172±455	30639±565	3858±258	391±24
mono -hydroxyl in B-ring							
kaempferol3-*O*-galactosylrutinoside (peak 48)	9913±230	29447±158	34507±409	34507±453	17290±104	349±8	50±3
kaempferol3-*O*-glucosylrutinoside (peak 51)	20651±125	118399±379	107556±379	96342±126	57396±214	461±16	36±2
kaempferol-3-*O*-galactoside (peak 54)	2357±95	3650±25	11120±98	9872±89	4744±96	1417±109	265±13
kaempferol-3-*O*-glucoside (peak 56)	1991±36	25962±97	11571±76	6882±202	5791±37	345±25	21±3
Summation	34912±486	177458±659	164754±962	147603±870	85221±451	2572±158	372±21
total flavonols	47163±1072	226147±1941	239490±2114	219018±1697	147157±1183	9711±620	763±45
Anthocyanidin (530 nm) (peak 60 and 61)	0.32±0.02	0.30±0.03	0.10±0.02	0.04±0.01	0.01±0.00	0.01±0.00	ND

ND, not detected; the mean ± SD of three independent analyses.

The accumulation patterns of quinic acid, GA derivatives, and HCA derivatives varied widely from the bud to fourth leaf (**Table 2**). Galloylquinic acid was the most predominant compound among the GA derivatives. The contents of the three gallic acid derivatives decreased remarkably with the development of leaves. For instance, the content of β-glucogallin and galloylquinic acid in the fourth leaf decreased to 10.37% and 11.69%, respectively, when compared to the bud. The accumulation of HCA derivatives was found to be developmentally regulated in leaf. The content was high in the first leaf, followed by the bud, and reduced significantly in the fourth leaf. The accumulation of GA and HCA derivatives in both stem and root were trace, but quinic acid was highly accumulated in the stem.

The amount of total catechins declined gradually from the bud to fourth leaf, but decreased significantly from the leaves and stem to root (**Table 2**). The contents of GC and C were low in the leaves, and EGC and EC gradually increased during the leaf development. In contrast, the amounts of galloylated catechins such as EGCG and ECG were predominant in the leaves and declined gradually with the development of leaves. Their contents in the fourth leaf reduced to 80.79% and 53.78%, respectively, compared to that with the bud. In terms of content and components, the catechins, especially galloylated catechins (EGCG and ECG) were abundant and diverse in both leaves and stems. On the contrary, the contents of catechins were very low in the roots. Galloylated catechins (EGCG and ECG) and tri-hydroxyl in B-ring catechins (GC and EGC) were found in trace, while only EC was detected in relatively larger extent.

Both UPLC-QQQ-MS/MS and *n-*butanol-HCl hydrolysis assays indicated that the content of PAs was much lower than the monomers of flavan-3-ol and shared the same trend with EGCG and ECG in the leaves at different developmental stages (**Table 2**). The content of PAs declined to 36.43% and 57.52% from the bud to fourth leaf, respectively.

Surprisingly, the total amount of PAs in the root was higher than in the leaves and stems. UPLC-QQQ-MS/MS findings displayed that the types of PAs were primarily procyanidin dimers and trimers in the leaves and stems (**Table 2**). However, the dimers (*m/z* 577) to pentamers (*m/z* 1553) of flavan-3-ol were detected in the root of tea plant (**Fig. S5**). Resulting anthocyanidins of PAs by *n*-butanol-HCl hydrolysis were primarily anthocyanidins with di-hydroxyl groups in the B-ring in the roots; whereas, anthocyanidins with mono-, di-, and tri- hydroxyl groups in the B-ring were simultaneously detected in the leaves (**Fig. S6**).

The contents of total flavonol derivatives were higher in the first and second leaves compared to the buds, and it changed dramatically in the leaves at different developmental stages (**Table 2**). For instance, the contents of flavonol derivatives in the first leaf were almost three times more than in the bud. But, the amount of flavonol derivatives in the leaves was markedly higher than the stem and root. Interestingly, the tri-hydroxyl in the B-ring flavonol was absent in the root.

Spectrophotometry analysis showed that the anthocyanin content was very low in the leaves and decreased gradually with the development of leaves (**Table 2**).

**Figure 4:**
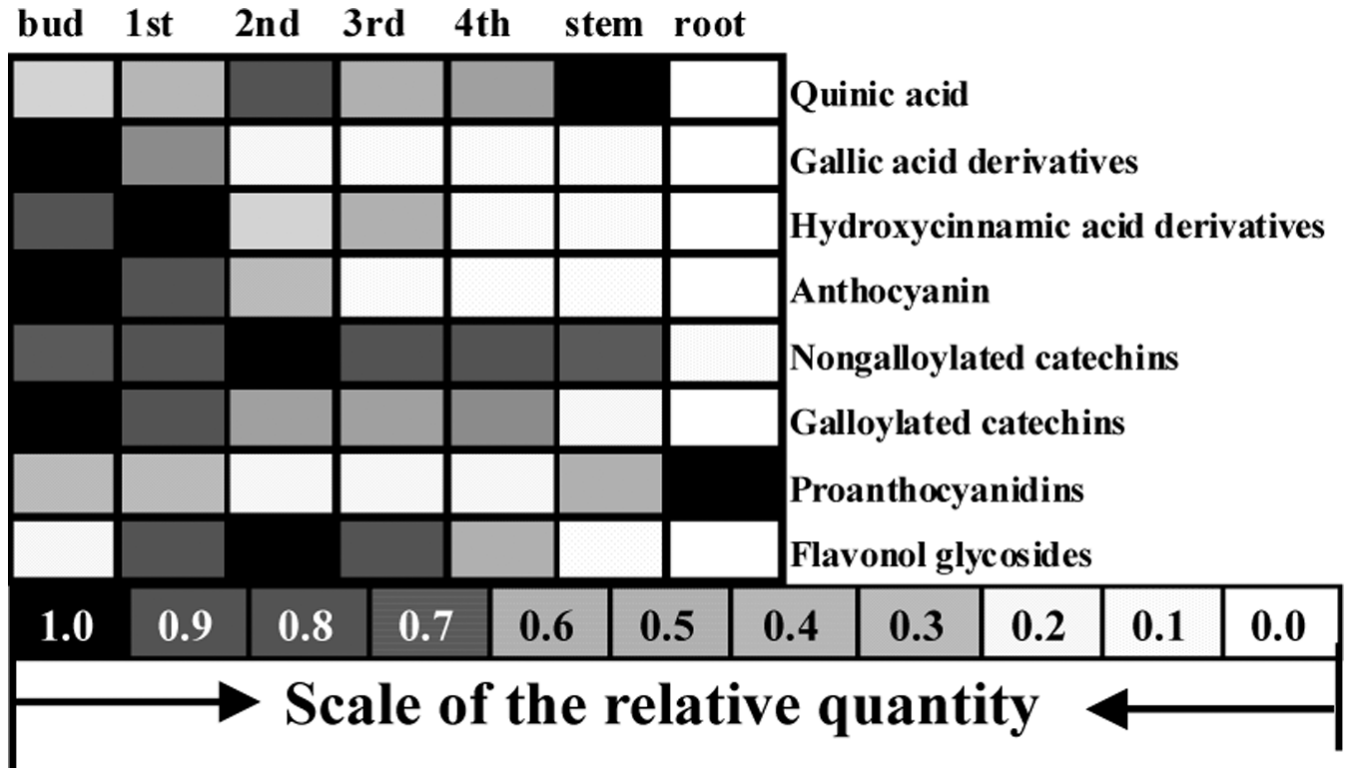
The relative quantities of phenolic compounds in different organs and leaves at different developmental stages were revealed according with those in table 2. Note: 1st, 1st leaf; 2nd, 2nd leaf; 3rd, 3rd leaf; 4th, 4th leaf.

Above all, the accumulation patterns of different phenolic compounds were variegated in different organs and leaves at different developmental stages. It suggested that different biosynthesis pathways gave rise to the phenolic compounds. These pathways were regulated along with the development of tea plant, although most phenolic compounds shared the main artery of flavonoid biosynthesis pathway (**Table 2** **,** **Fig. 4**).

### The Analyses of Gene Expression and Enzymatic Activity

The structural and regulator genes related to the metabolism of phenolic compounds in tea plant were very few in the NCBI database. From the transcriptome database and NCBI database, 63 predicted structural and regulator genes related to the metabolism of phenolic compounds were screened out and obtained by high-throughput illumina (**Table S1**).

**Figure 5:**
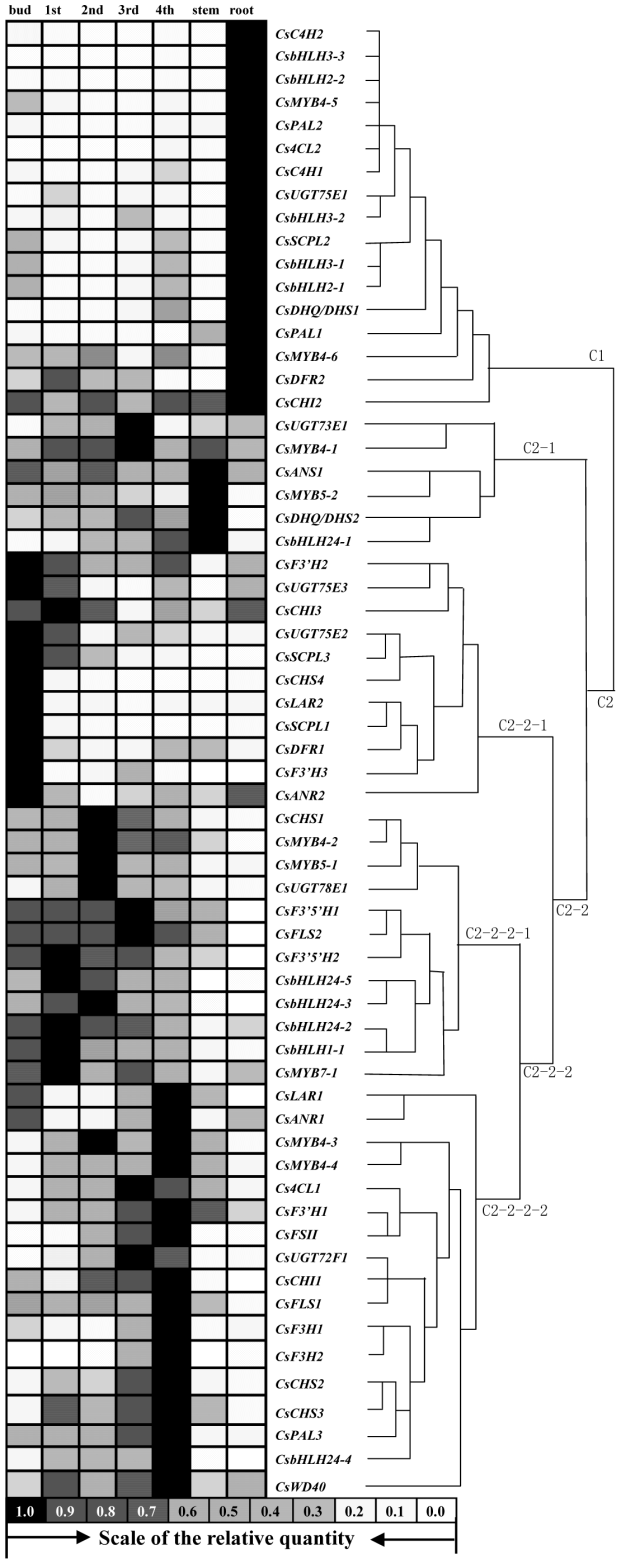
The relative expression rate of genes involved in biosynthetic pathway of phenolic compounds in different organs and leaves at different developmental stages were showed on the left, and the dendrogram represented the hierarchical clustering analysis using the between-groups linkage method performed with gene expression data on the right. Note: 1st, 1st leaf; 2nd, 2nd leaf; 3rd, 3rd leaf; 4th, 4th leaf.

To acquire the key genes involved in the metabolism of phenolic compounds, the expression patterns of these predicted genes in the leaves at different developmental stages and different organs were investigated, and a representative hierarchical cluster of the gene expression data was performed using the between-groups linkage method (**Fig. 5**). Based on the similarity of their expression patterns, the 63 screened genes were classified into two main groups: C1 and C2; and four subgroups were clustered further in C2 group.

**Figure 6:**
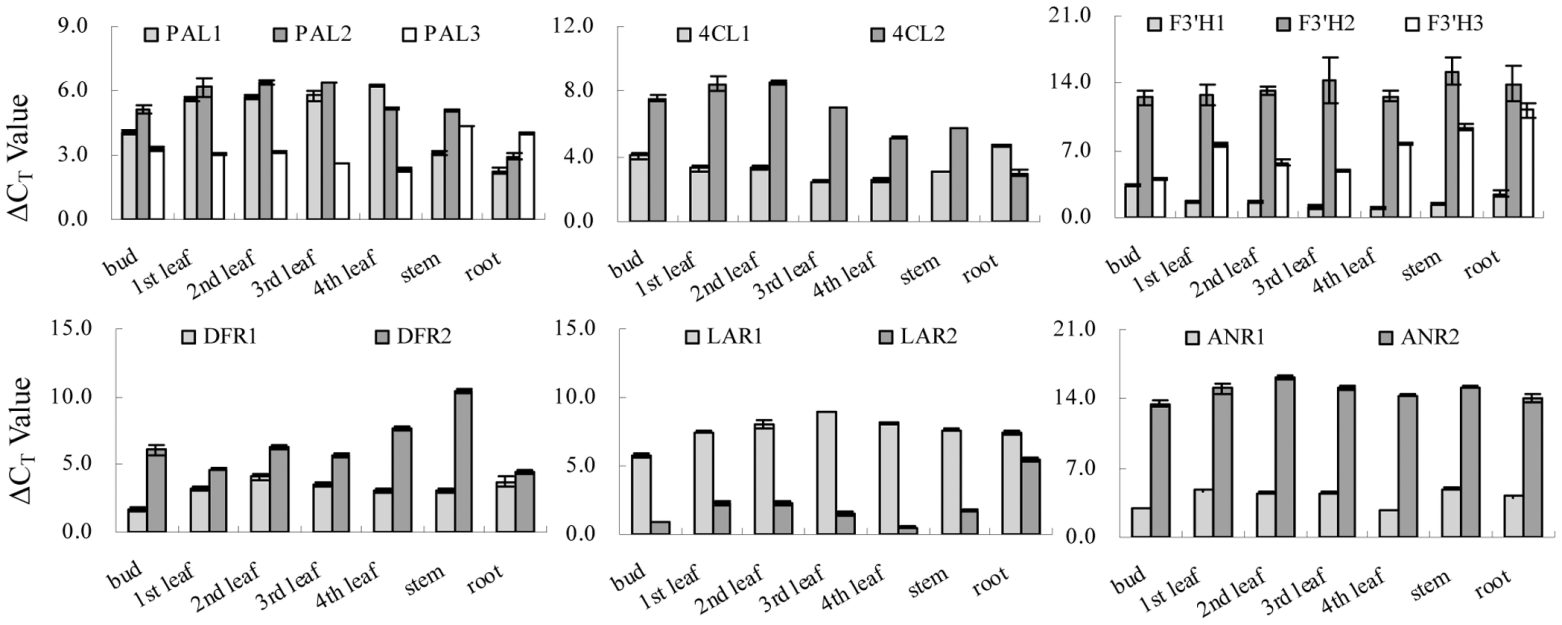
ΔCt values of gene expressions in multi-gene family. The genes involved in the metabolic pathway of phenolic compounds in different organs and leaves at different developmental stages.

The genes in group C1 had high relative expression level in the root and low in the bud and leaves. For instance, the structural genes *CsPAL1*, *CsPAL2,* and *Cs4CL1* were highly expressed in the root; and *CsDFR2* had low relative expression level in the bud and leaves compared with other genes in the multi-gene family (**Fig. 6**). Further verification is required to understand whether these expression patterns are relevant to the accumulation of PAs in high levels in the root (**Fig. 4**).

*CsMYB4-5* and *CsMYB4-6* in group C1 were clustered into subgroup 4 in 27 *C. sinensis* R2R3-MYBs subgroups; and *CsbHLH2-1* and *CsbHLH2-2* in group C1 were clustered into subfamily 2 in 9 *C. sinensis bHLHs* subfamilies in a recent study [36]. The MYB proteins of subgroup 4 were shown to be the representative factors for the phenolic acid metabolism and lignin biosynthesis by interacting with bHLH proteins [50], [51], and the bHLH proteins of subfamilies 2 and 24 were predicted to be involved in the flavonoid metabolism [36]. It suggested that the high expression levels of *CsMYB4-5*, *CsMYB4-6*, *CsbHLH2-1* and *CsbHLH2-2* (**Fig. 5**) might lead to low phenolic acid levels in the root (**Fig. 4**).

The expression patterns of several subgroup genes in group C2 revealed the development-dependent character (**Fig. 5**), which might be responsible for the difference in accumulation pattern of phenolic compounds in the leaves at different developmental stages (**Fig. 4**).

The expression patterns of the genes in subgroup C2-2-1 showed high expressions in the bud, which decreased significantly with the development of the leaves (**Fig. 5**). These observations were in accordance with the accumulation patterns of gallic acid derivatives, HCA derivatives, anthocyanidins, and galloylated catechins (**Fig. 4**). Some genes in this subgroup had proved to be involved in the biosynthesis of gallic acid derivatives and galloylated catechins. For instance, CsUGT75E2 and CsUGT75E3 could be gathered in group L of the phylogenetic tree together with Arabidopsis UGT75B1 (data were not shown); and the latter had been identified to catalyze the formation of glucose esters of the benzoates at the carboxyl group of the aglycone [52]. Glucose esters of aglycones (such as β-glucogallin) were considered as the biosynthetic intermediates, and the high energy of the glucose ester might drive the transfer of aglycone to a further acceptor [37]. Although the expression patterns of *CsUGT75E2* and *CsUGT75E3* were similar to that of UGGT activity (**Figs. 5** **and** **7**), the function of these genes should have been identified. Likewise, *CsSCPL* might involve in the galloylation of catechins in tea plant (Liu et al. 2012), and further confirmation is needed to observe whether or not the EGGT is expressed by *CsSCPL1* or *CsSCPL3*.

**Figure 7:**
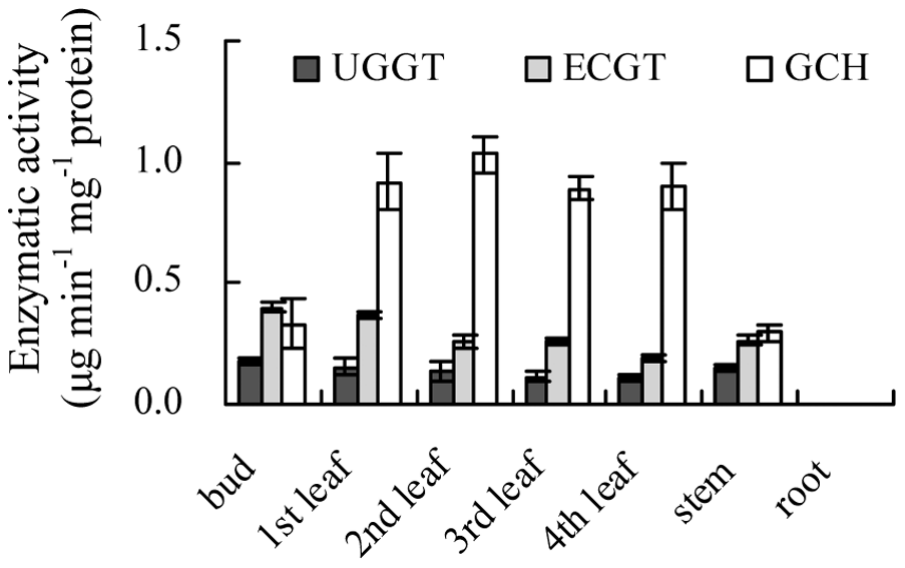
Analyses of enzymatic activity involved in catechins galloylated and hydrolysis biosynthesis in different organs and leaves at different developmental stages. The enzyme activity unit was defined as the amount of product formed by one milligram of enzyme catalyzed in one minute (µg min^−1^ mg^−1^ protein); The UGGT activity was assayed with the substrates gallic acid. The ECGT activity was assayed with the substrates EGC and β-glucogallin. The GCH activity was assayed with the substrate EGCG.

The level of gene expressions in group C2-2-2-1 were higher in the first and second leaves (**Fig. 5**), which were in accordance with the accumulation patterns of flavonol derivatives and nongalloylated catechins (**Fig. 4**). It suggested that the expressions of the structural genes such as *CsCHS1*, *CsF3’5’H1*, *CsF3’5’H2*, *CsFLS2* and *CsUGT78E1*; and regulator genes such as *CsMYB5-1*, *CsMYB4-2*, *CsMYB7-1*, *CsbHLH24-5*, *CsbHLH24-3* and *CsbHLH24-2* might be responsible for the biosynthesis of flavonol derivatives and nongalloylated catechins. Likewise, the low expression of *CsF3’5’H1*, *CsF3’5’H2*, and *CsFLS2* in the root might directly lead to the lack of tri-hydroxyl group compounds such as catechins (EGCG, EGC and GC), flavonols (myricetin), and anthocyanidins (delphinidin) in the B-ring in root (**Table 2** **,** **Fig. 5** and **Fig. S6**). Published literature showed that the subgroup 7 of the MYB gene family participated in the regulation of flavonol biosynthesis [53], and the subfamily 24 of the bHLH gene family was identified as the regulators in the flavonoid or anthocyanin metabolism [54]. The high expression of *CsMYB7-1* and some *CsbHLHs* such as *CsbHLH24-5*, *CsbHLH24-3* and *CsbHLH24-2* in the tender leaf might enhance the accumulation of flavonol derivatives associating with the above structural genes (**Figs. 4** **and** **5**).

Some structural genes related to the phenylpropanoid pathway and flavonoid synthetic pathway in group C2-2-2-2 had higher expressions level in the mature leaves. Further studies are required to understand their functions.

Interestingly, the hierarchical cluster analysis based on the similarity of the expression patterns did not show the real transcriptional level of genes. The C_T_ value might be used to show the real transcriptional level of genes and compare breadth-wise the different expressions of multi-gene family genes in a sample. Different transcriptional levels in some multi-gene family such as *PAL1/PAL2/PAL3*, *4CL1/4CL2*, *F3’H1/F3’H2/F3’H3*, *DFR1/DFR2*, *LAR1/LAR2*, and *ANR1/ANR2* were observed (**Fig.** **6** **)**, which suggested that these genes were from the multi-functional gene family.

Besides anabolism, the accumulation of phenolic compounds could be influenced by catabolism. The accumulation of nongalloylated catechins were considered to be aroused from the hydrolysis of galloylated catechins. The activities of enzymes involved in the metabolism of galloylated catechins were investigated in the leaves at different developmental stages and different organs (**Fig. 7**). During leaf development, the activity of GCH increased; and it was in contrast with UGGT and ECGT, which lead directly to an increase in nongalloylated catechins (EC and EGC) (**Table 2** **,** **Figs. 4** **and** **7**).

In addition, no obvious activities of UGGT, ECGT, and GCH were detected in the root. The lower contents of GA and βG (the substrates for galloylation of catechins), coupled with lower activities of UGGT and ECGT, possibly were attributed to the absence of the galloylated catechin (ECG and EGCG) in the roots.

## Discussion

For the past few years, flavonoids have attracted due attention and revealed diverse pharmacological activities and biological functions through a good number of outstanding researches [16], [22], [28], [55]–[57].

There were varieties of phenolic compounds in tea plant. The polyphenols in fresh tea leaves and other tea products included flavonols (*O*-glycosylated flavonols and acylated glycosylated flavonols), flavan-3-ols (catechins, methylated catechins, and PAs), phenolic acid ramifications, and flavones [18]. Simultaneous determination of polyphenols may comprehend the synthetic diversity and regulation-complexity of polyphenols biosynthesis in tea. HPLC with UV detection has extensively been used for the analysis of phenolic compounds, especially catechins (flavan-3-ols) [29], [58]–[60]. However, the inability to detect few compounds simultaneously with catechins by HPLC has created the need for other analytical options. For example, quinic acid did not have an absorption spectrum in the UV-Visible spectrometer. Likewise, ECG and quercetin 3-*O*-galactosylrutinoside could not be separated effectually using a HPLC column; and flavones and PAs were difficult to be precisely quantified for their low content. Some of them could not be identified due to the lack of standard substances as well. MS-MRM mode could dramatically impact on improving the simultaneous determination of those phenolic compounds in tea plant. In this study, 50 polyphenols were quantitatively analyzed by means of LC-TOF-MS and UPLC-QQQ-MS/MS (**Table 1** **,** **Figs. 2** **and** **3**), and 29 phenolic compounds were qualitatively and quantitatively analyzed simultaneously by UPLC-QQQ-MS/MS (**Table 2**).

Several studies have focused on the tissue-specific, development-dependent, external stimulation (including sugar, hormones, drought, wounding, and UV-B irradiation response) to accumulation of phenolic compounds and related gene expression in tea plant [43], [44], [61]–[68]. The study results obtained were in agreement with the previous researches. The accumulation of phenolic compounds in leaves were developmentally regulated, and the content of most phenolic compounds such as gallic acid derivatives, HCA derivatives, galloylated catechins, PAs, and anthocyanidin were highest in the bud or first leaf and declined gradually along with the development of leaves. Some other phenolic compounds such as quinic acid, nongalloylated catechins, and flavonol derivatives were very low in the bud but increased markedly along with the development of leaves.

The expression patterns of the basic genes related to the accumulation of total polyphenols at different stages of tea leaf development and their relationship with catechins concentration have been investigated in many studies. However, the understanding on the phenolic compound metabolic flux in tea plant is still scanty as only a fewer genes have been logged in the NCBI database. In the recent *C.sinensis* transcriptome database, the *C. sinensis* phylogenetic trees have been constructed for R2R3-MYB and bHLH regulatory proteins using the previous *Arabidopsis* data with further classification into 27 subgroups and 32 subfamilies [36].

A hierarchical clustering analysis suggested that the screened 63 genes were classified into two main groups - C1 and C2. The expression patterns of genes in C2 subgroups were development-dependent, and the expression patterns of genes in C2-2-1 and C2-2-2 subgroups probably were concerned with the accumulation of phenolic compounds in leaves. For instance, the expression of *CsUGT75E2*, *CsUGT75E3*, *CsSCPL1*, and *CsSCPL3* were consistent with the accumulation of galloylated catechins, while these genes might be involved in the biosynthesis of galloylated catechins [37]. *CsMYB7-1* in C2-2-2-1 group was another example. The genes in subgroup7 might participate in the regulation of flavonol biosynthesis [53], [69]. The expression of *CsMYB7-1* had high accumulation of flavonol derivatives. The subfamilies 2, 5, and 24 of the *bHLH* gene family in plants were identified as the regulators in the metabolism of flavonoid or anthocyanin [54]. The expression patterns of some *CsbHLHs* were similar to that of *CsMYB7-1*.

The total amount of PAs was highest in the root. But the root lacked gallic acid derivatives, galloylated catechins, and tri-hydroxyl in the B-ring flavonol and flavan-3-ols (**Table 2** **and** **Fig. 4**). The *CsF3’5’Hs* were responsible for tri-hydroxyl group in the B-ring phenolic compounds; and *CsUGT75E2*, *CsUGT75E3*, *CsSCPL1*, and *CsSCPL3* might have attributed for the galloylation of catechins. These genes were absent in the root (**Fig. 5**). R2R3-MYBs of subgroup 4 were shown to represent the phenolic acid metabolism and lignin biosynthesis. *CsMYB4-5* and *CsMYB4-6* were highly expressed in roots, which suggested that they might be closely related to repress phenolic acid biosynthesis.

The biosynthesis and regulation of PAs were quite well understood in *A. thaliana*, *M. truncatula*, and other model plants; and the study on relevant regulatory genes were carried out. MYB TFs that were shown to involve in the regulation of PAs biosynthesis included the following: TRANSPARENT TESTA2 from *Arabidopsis thaliana* [70], MYBPA1 and MYBPA2 from *Vitis vinifera* [71], and DkMyb4 from the fruit of *Diospyros kaki* [72]. Recently, it had been reported that the *M. truncatula* PAs regulator, an R2R3-MYBs transcription factor of subgroup 5 acted as a key regulator of PAs biosynthesis rather than anthocyanin biosynthesis; and it positively regulated *CHS*, *F3H*, *ANS*, and *ANR* through a probable activation of WD40-1 [22]. MATE1, a precursor transporter involved in the biosynthesis of PAs had been isolated and characterized from the model legume *M. truncatula* 23,73,74. Nevertheless, relatively little is known about the pivotal structural gene of PAs polymerization from monomer flavan-3-ol. It was not clear that why PAs polymerization took place in the root rather than in the leaves of tea plant. Galloylation of catechins at position 3 of the C-ring could possibly interfere with the polymerization in the leaves.

PAs were rapidly up-regulated by stresses such as pathogen infection, wounding and herbivory [75]–[77]. The high accumulation of PAs is to protect plants including tea plant from being attacked by microbial pathogens, insect pests and larger herbivores [78].

It is most remarkable that accumulation profile of the nongalloylated catechins might be influenced by synthesis and catabolism. The level of ANR expression is not absolutely consistent with the accumulation of epicatechins in this paper. The results of Pang showed that the recombinant ANR1 and ANR2 proteins produce EC, C, EGC and GC [79]. In our experiment (data not shown), however, utilizing partly purified ANR enzyme from tea plant, only EC and EGC were detected, without any trace of C and GC. The results suggested the function of recombinant proteins may be not consistent with the enzyme in tea plant, or there may be another ANR gene in tea plant. In our transgenic experiment (data not shown), the level of ANR expression is consistent well with the content of DMACA-stained compounds. In fact, the accumulation of EC is affected by biosynthesis, hydrolysis of galloylated catechins [38], polymerization [24] and galloylation [37] jointly.

## Conclusion

Of the 50 phenolic compounds identified, 29 were quantified based on their fragmentation behaviors. The accumulation of phenolic compounds in the tea plant was developmentally regulated in bud, leaves, and root; and the content was higher in younger leaves. The expression patterns of genes in C2-2-1 and C2-2-2-1 groups were probably responsible for the development-dependent accumulation of phenolic compounds in the leaves. Further researches may help to understand the expression patterns and functions of various genes involved in the biosynthesis and/or metabolism of different compounds in tea plant and the reason for the variation in their content in different growth stages and also in different organs.

## Supporting Information

Figure S1 **MS/MS of phenol acids.**
(TIF)

Figure S2 **MS/MS of catechins derivatives.**
(TIF)

Figure S3 **MS/MS of PAs.**
(TIF)

Figure S4 **MS/MS of flavonols derivatives.**
(TIF)

Figure S5 **Chromatogram of HPLC-TOF-MS for phenolic compounds in tea plant: (A) Total ion chromatography of leaf, (B) Total ion chromatography of stem, and (C) Total ion chromatography of root.** The sample preparation methods are described in the Materials and Methods. The peaks were listed in Table 1.
(TIF)

Figure S6 **HPLC-TOF-MS analyses of resulting anthocyanidin of PAs by butanol-HCl hydrolysis in tea.** (A), (B), and (C) represent HPLC of resulting anthocyanins of PAs by butanol-HCl hydrolysis in root, stem, and leaf, respectively; and (D), (E), and (F) represent MS of peak 1, 2, and 3, respectively.
(TIF)

Table S1 **Sequences of primers used to amplify genes involved in phenolic compounds biosynthesis in** ***Camellia sinensis*** **(L.).**
(DOC)
