# Supplementary figures and images for: Tissue-Specific, Development-Dependent Phenolic Compounds Accumulation Profile and Gene Expression Pattern in Tea Plant [Camellia sinensis]
Source: PLoS One. 2013 Apr 30;8(4):e62315. doi: 10.1371/journal.pone.0062315 (PMC3639974; doi:10.1371/journal.pone.0062315)

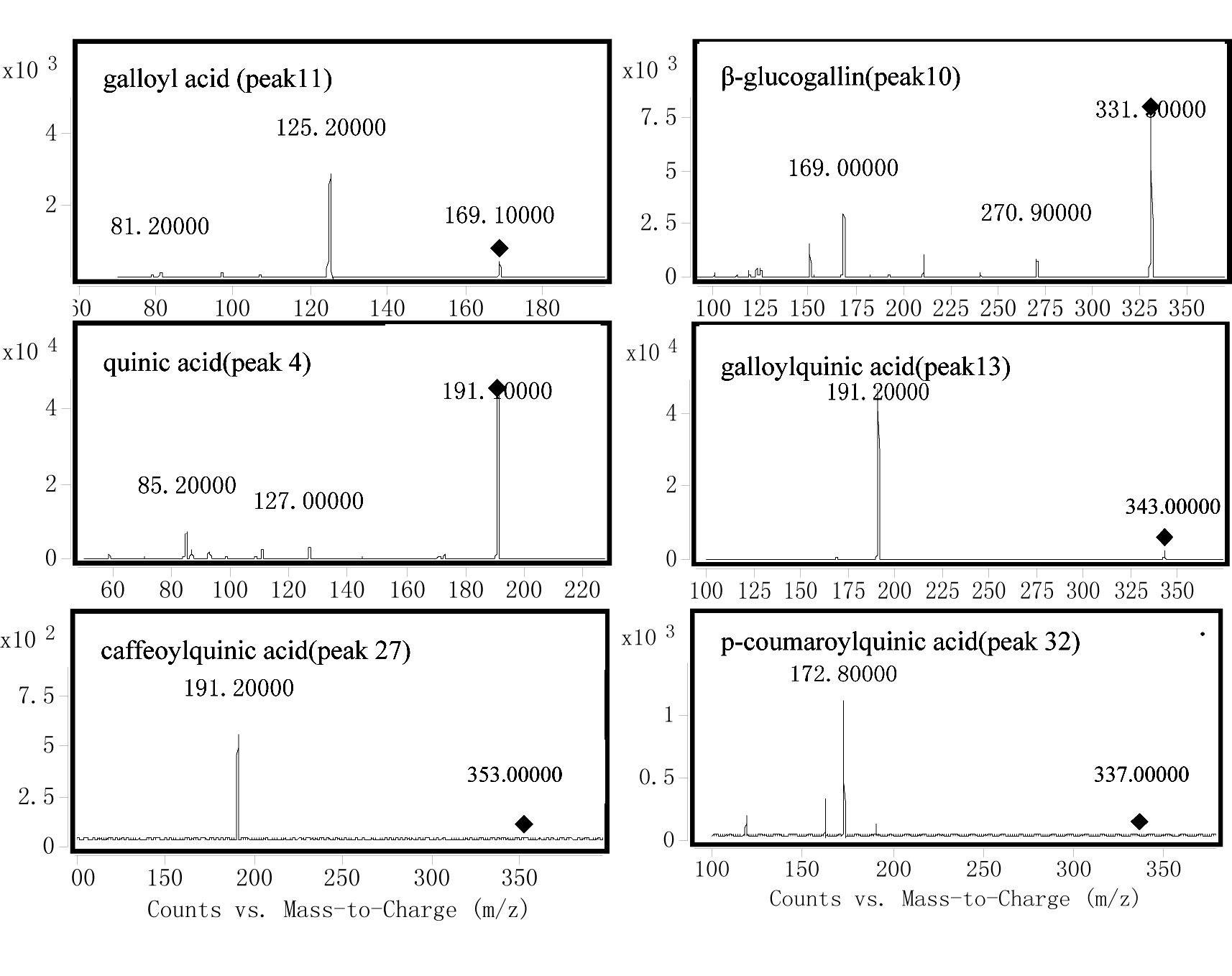

Supplement: Figure S1 — MS/MS of phenol acids. (TIF) [file pone.0062315.s001.tif]

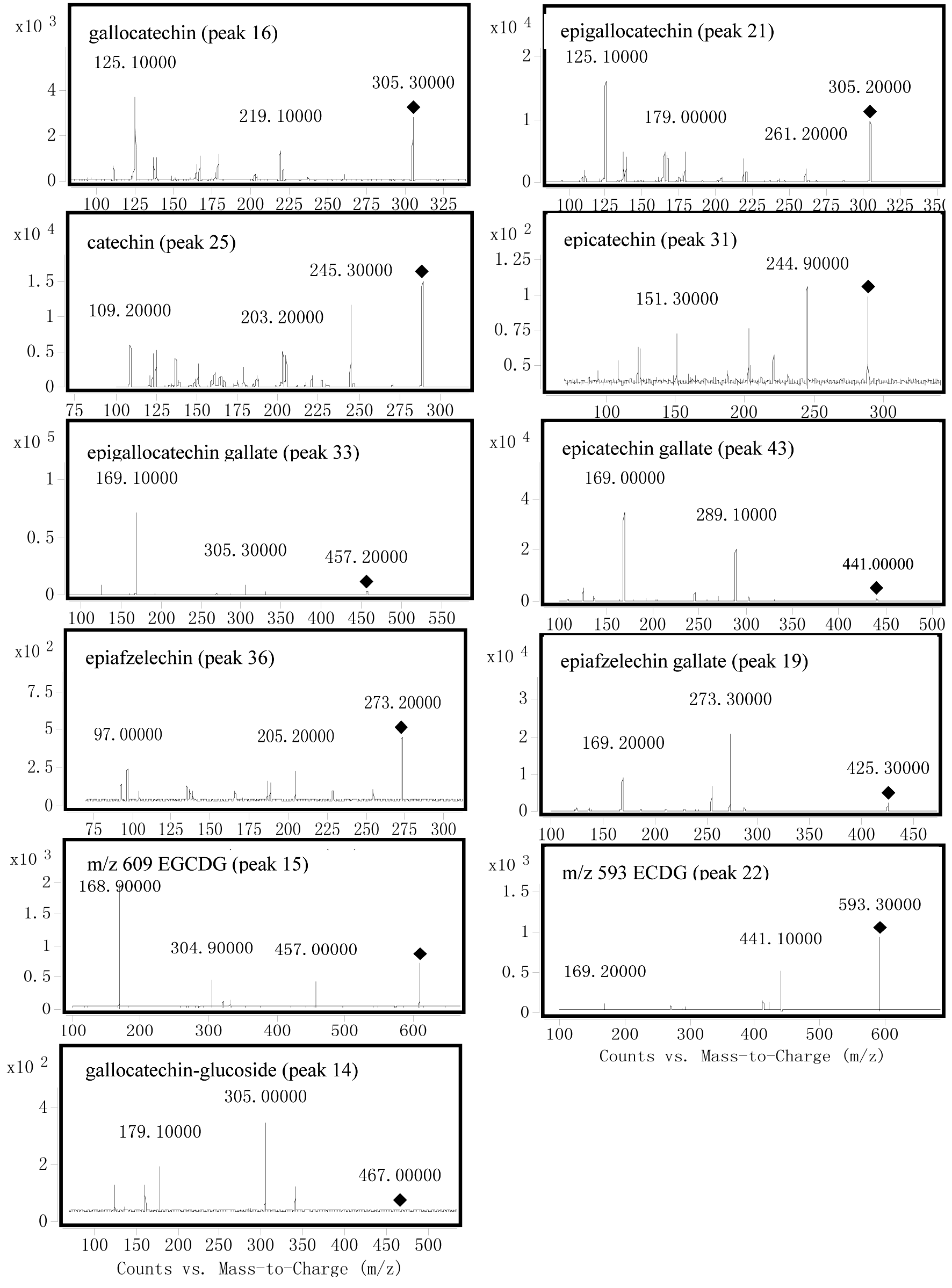

Supplement: Figure S2 — MS/MS of catechins derivatives. (TIF) [file pone.0062315.s002.tif]

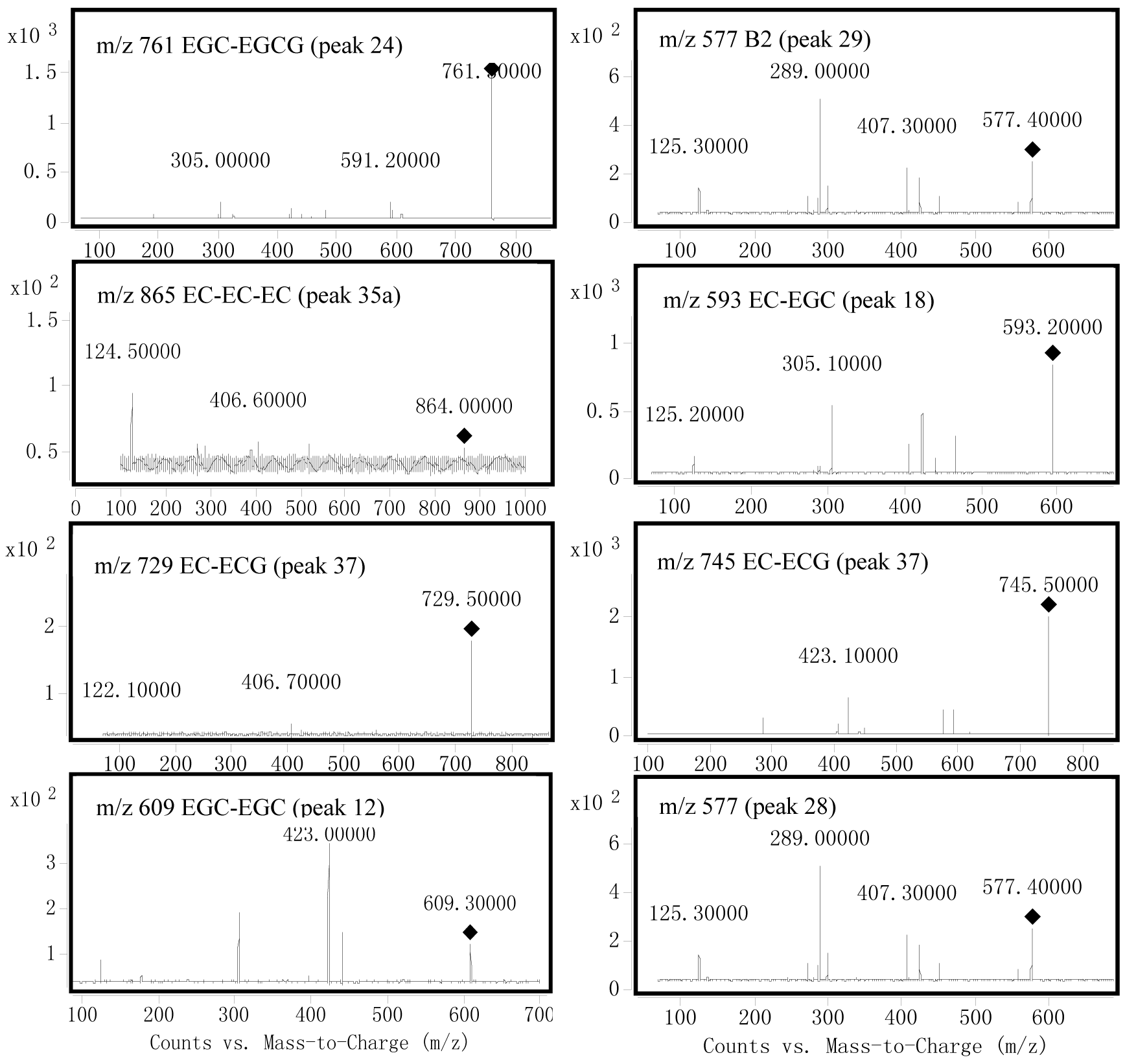

Supplement: Figure S3 — MS/MS of PAs. (TIF) [file pone.0062315.s003.tif]

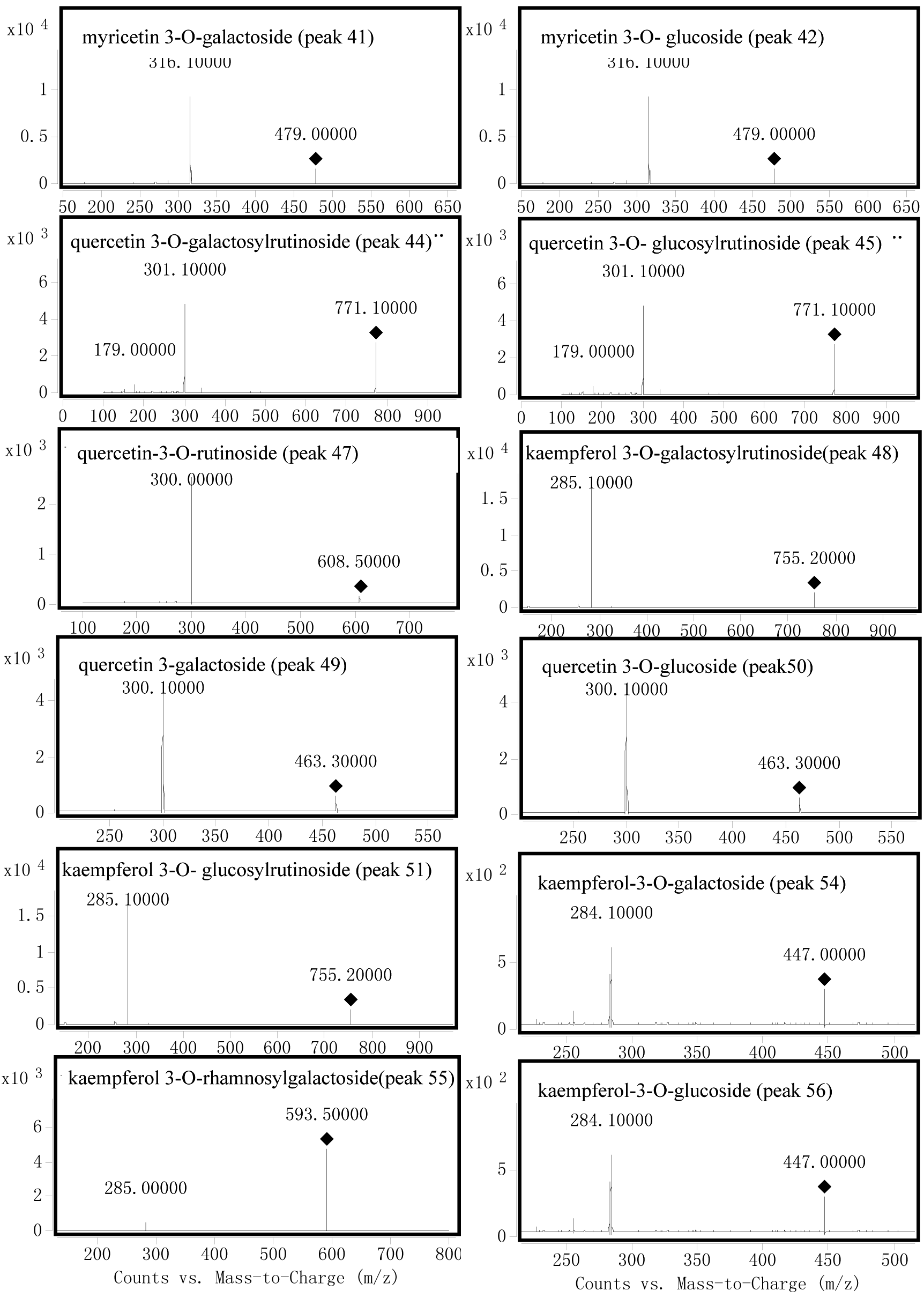

Supplement: Figure S4 — MS/MS of flavonols derivatives. (TIF) [file pone.0062315.s004.tif]

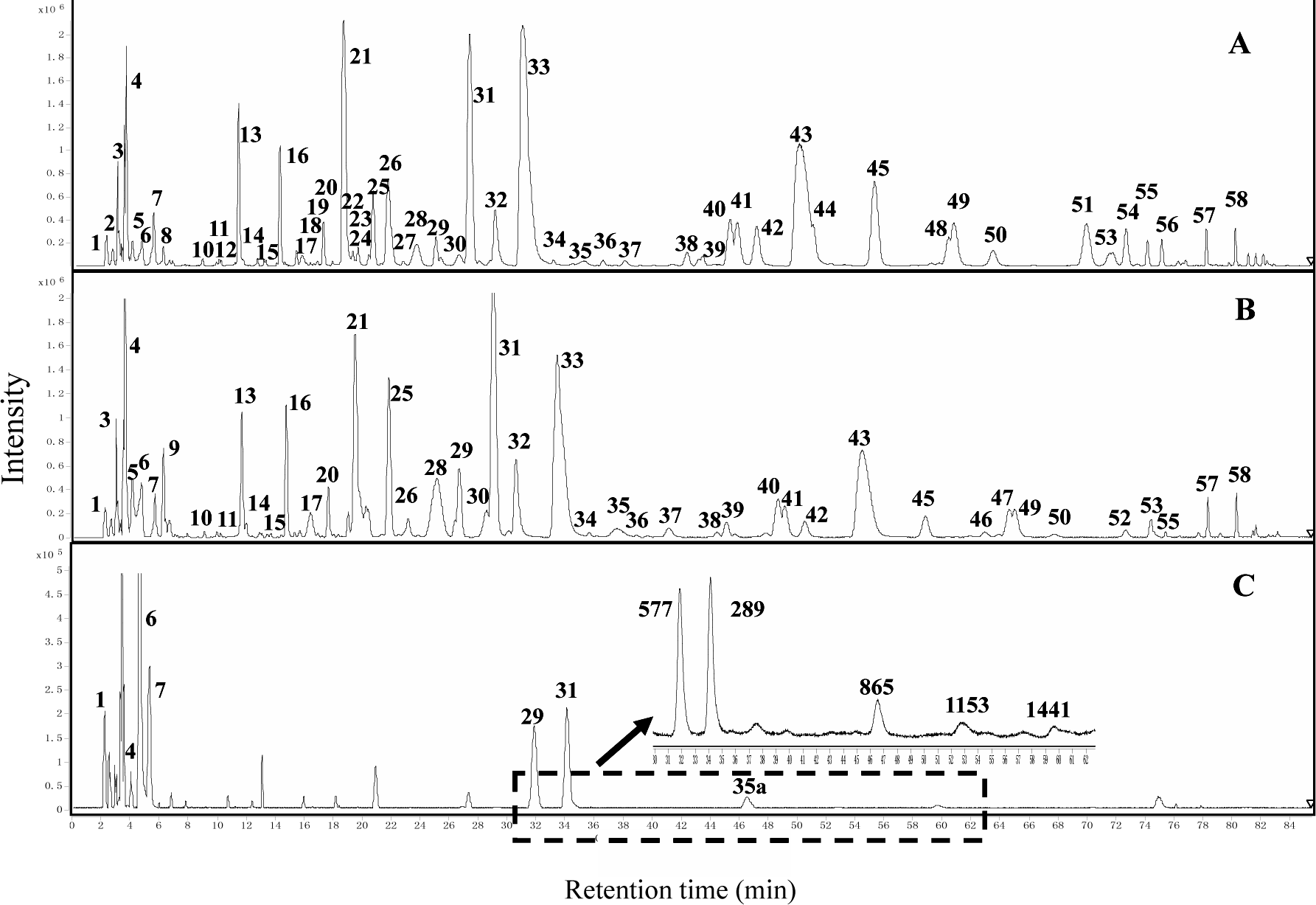

Supplement: Figure S5 — Chromatogram of HPLC-TOF-MS for phenolic compounds in tea plant: (A) Total ion chromatography of leaf, (B) Total ion chromatography of stem, and (C) Total ion chromatography of root. The sample preparation methods are described in the Materials and Methods. The peaks were listed in Table 1. (TIF) [file pone.0062315.s005.tif]

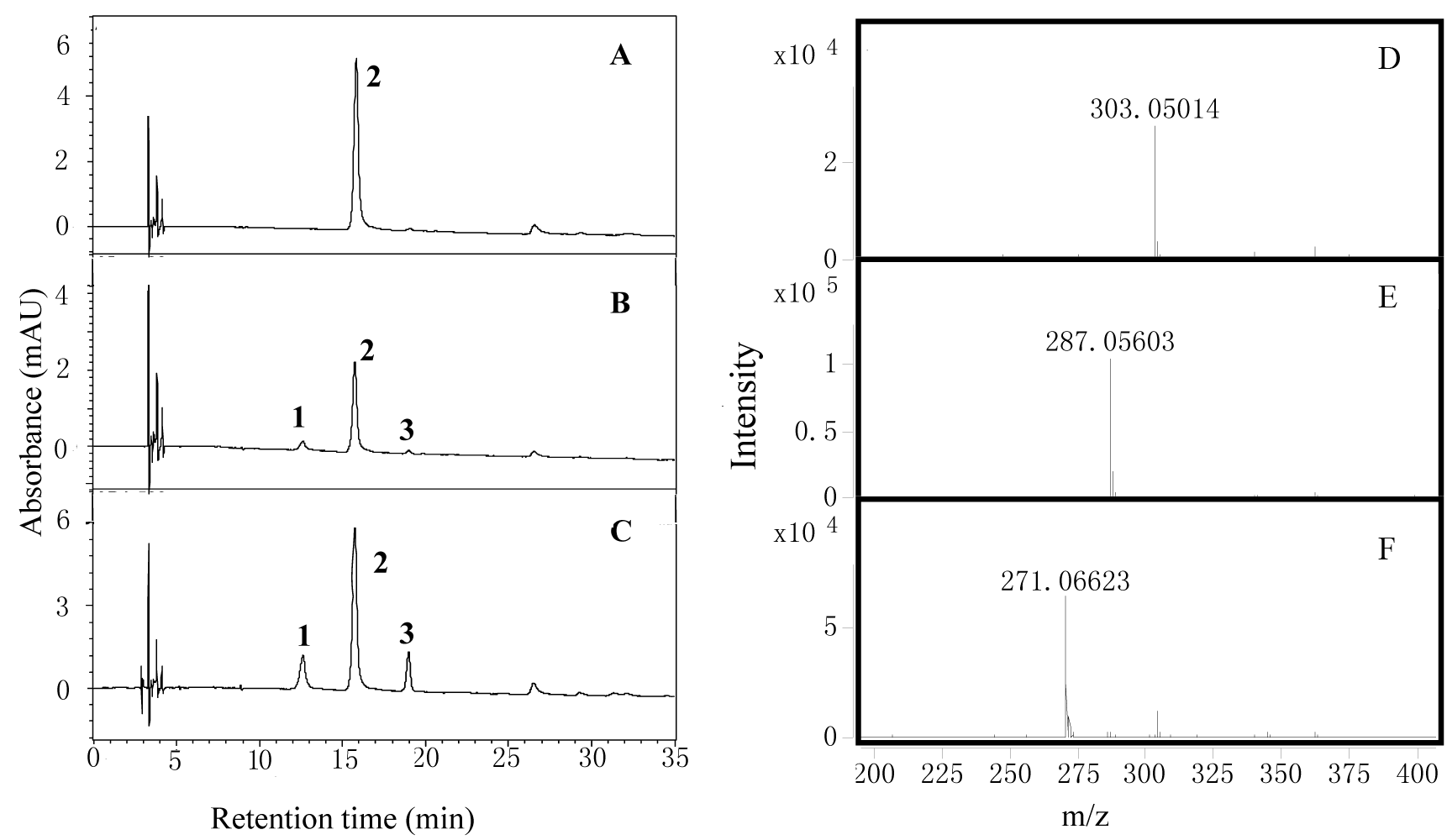

Supplement: Figure S6 — HPLC-TOF-MS analyses of resulting anthocyanidin of PAs by butanol-HCl hydrolysis in tea. (A), (B), and (C) represent HPLC of resulting anthocyanins of PAs by butanol-HCl hydrolysis in root, stem, and leaf, respectively; and (D), (E), and (F) represent MS of peak 1, 2, and 3, respectively. (TIF) [file pone.0062315.s006.tif]
