# Supplementary material for: Tissue-Specific, Development-Dependent Phenolic Compounds Accumulation Profile and Gene Expression Pattern in Tea Plant [Camellia sinensis]
Source: PLoS One. 2013 Apr 30;8(4):e62315. doi: 10.1371/journal.pone.0062315 (PMC3639974; doi:10.1371/journal.pone.0062315)
Supplement: Table S1 — Sequences of primers used to amplify genes involved in phenolic compounds biosynthesis in Camellia sinensis (L.). (DOC) [file pone.0062315.s007.doc]

| **Table S1 Sequences of primers used to amplify genes involved in phenolic compounds biosynthesis in *Camellia sinensis* (L.)** | | |
| --- | --- | --- |
| Gene name | GenBank accession No.  or transcriptome dataset ID | Primer sequences |
| *GAPDH* | XM 002263109 | F5’-TTGGCATCGTTGAGGGTCT-3’  R5’-CAGTGGGAACACGGAAAGC-3’ |
| *CsDHQ/DHS1* | tie_GLEAN_10008767 | F5’-CACTGGATTACCCGAATGTTCT-3’  R5’-ACCATTGGATTTCTGAAGTAGCA-3’ |
| *CsDHQ/DHS2* | tie_GLEAN_10041890 | F5’-CTGGTGGATTAGTGCCAGAAGA-3’  R5’-CAGTCAATGGCTCAGGTGGG-3’ |
| *CsPAL1* | D26596 | F5’-TCTgCCAAgCCgTAgATTTgA-3’  R5’-AACgCCCATAgTTAgAACgCg-3’ |
| *CsPAL2* | tie_GLEAN_10033588 | F5’-ACATTCAGCAACAAGAGCCG-3’  R5’-CAATGTAAGACAAGGGGACGAG-3’ |
| *CsPAL3* | Singletons 109363 | F5’-TCCAAAGCACCGAGCAACA-3’  R5’-CACTATCCACCACTGTGAGCAAG-3’ |
| *CsC4H1* | AY641731.2 | F5’-ATAGAATGGGGCATAGCAGAA-3’  R5’-TTGGAGGTAGGGGAGTTTGT-3’ |
| *CsC4H2* | cam_GLEAN_10005413 | F5’-TTGCCGTTTGGTATGG-3’  R5’-AGCTGAACTGCCCTCC-3’ |
| *Cs4CL1* | DQ194356.1 | F5’-GAGCGAAATCCCGACAGT-3’  R5’-TCCAGCAACGCCCCTAT-3’ |
| *Cs4CL2* | Singletons 47605 | F5’-GCTGCTGTTGTCCCGATGA-3’  R5’-TTGCCAGACGGTGCCTTT-3’ |
| *CsCHS1* | D26593.1 | F5’-GGCAATCAAAGAATGGGG -3’  R5’-ATGGGCGAAGACCGAGT-3’ |
| *CsCHS2* | D26594.1 | F5’-GGATGTTCCTGGGCTTATTTC -3’  R5’-GGTCTTGAGTCCTTTCTTTGCTGA -3’ |
| *CsCHS3* | D26595.1 | F5’-ATCAGCCACCACCCTAAACA -3’  R5’-CTCCACCTTATGCTCGCTATTA -3’ |
| *CsCHS4* | Singletons 9897 | F5’-GTTGCCAAAGACATTGCTGA-3’  R5’-CTTCTGTGAGGCGTCCATC-3’ |
| *CsCHI1* | DQ904329.1 | F5’-GTTGAGACTGAACCCAAGACC-3’  R5’-TCGCACCAGCATTCCTAC-3’ |
| *CsCHI2* | DQ120521.2 | F5’-CCCTGGCGGTTCTATTC-3’  R5’-TGACTCCAACACTGCTTCTG-3’ |
| *CsCHI3* | GT012166.1 | F5’-CGTTACAGGTCCCTTTGAGA-3’  R5’-ATAAGACAAGTGGGTCAGTGG-3’ |
| *CsF3H1* | AY641730.1 | F5’-CTACTCAAGATGGCCCGACAA-3’  R5’-ACAACACCTCCAGCAACTTGC-3’ |
| *CsF3H2* | cam_GLEAN_10017094 | F5’-ACGAATCCACCGAGGACTGC-3’  R5’-ACTGCGCCACTACCAACCGA-3’ |
| *CsF3’H1* | GQ438849 | F5’-ACCTTTCGACTTCACCCATCAAC-3’  R5’-TAACTGGACCATACGCAACCCTA-3’ |
| *CsF3’H2* | tie_GLEAN_10006667 | F5’-GGGAGCACATTTGACAGC-3’  R5’-CATTTTCACCCTCGCATC-3’ |
| *CsF3’H3* | Singletons 5418 | F5’-CGACTTCTTAGACTTACAAGGATAC-3’  R5’-ACCGAGCACGAGGTCAT-3’ |
| *CsF3’5’H1* | AY945842 | F5’-CTCAATCTTCCCAGAGTCGC-3’  R5’-CAACAAGCACAATCCCCAT-3’ |
| *CsF3’5’H2* | tie_GLEAN_10020759 | F5’-GCAGATTATGGACCTAGATGGAAG-3’  R5’-GCTCACGGCGACAACTCAA-3’ |
| *CsDFR1* | AB018685 | F5’-ATTgGCAgAgAAAgCAgCAT-3’  R5’-gTgATTAggCTTggTgggAA-3’ |
| *CsDFR2* | Singletons 112402 | F5’-AATGGTTATGGTGGTGGCG-3’  R5’-CGAAATTCATGGGAGTGGC-3’ |
| *CsLAR1* | EF205148.1 | F5’-GCAGCCTCTACCCTGATGA-3’  R5’-GGCAACGAATTGACTCCC-3’ |
| *CsLAR2* | cam_GLEAN_10033377 | F5’-AAGCGAATGGTGAGGAGG-3’  R5’-GGATGACATCAGCAGGGTG-3’ |
| *CsANS1* | AY830416.1 | F5’-GGCCACAAGTGCCTACAATTG-3’  R5’-CCCATGATTCACCAAATGCA-3’ |
| *CsANR1* | AY641729.1 | F5’-GCGAAGTTGATCCTCTCGTC-3’  R5’-AACCACATCGTCAAGTGAACA-3’ |
| *CsANR2* | AY169404.1 | F5’-TTTAAAAGCCTGCGCGAAAG-3’  R5’-ATGACAAGCCCCGTTCCAT-3’ |
| *CsFLS1* | EF205150.1 | F5’-ATACAGGGGAGTGACAGAGGA-3’  R5’-ATTGGGGACAAGTAAAGTGAGA-3’ |
| *CsFLS2* | FJ577509 | F5’-TCCACCAAAATACAAGACCAAG-3’  R5’-CAGTGAAACGAGTGAGTGAAGAA-3’ |
| *CsFSII* | FJ169499 | F5’-CCCGTGAACTACACTGCT-3’  R5’-GTTCCTGATAACCCACATAA-3’ |
| *CsUGT75E3* | cam_GLEAN_10005292 | F5’-GCTACAGAAAATGGCGAGAA-3’  R5’-GTGAAACGAAGCACCCTAAA-3’ |
| *CsUGT75E2* | tie_GLEAN_10036715 | F5’-TTGCCAATGCCAAAGCG-3’  R5’-CAATGACGGGTGCGAAAG-3’ |
| *CsUGT75E1* | cam_GLEAN_10009902 | F5’-CCAGCAGCCCTTTTCCGTA-3’  R5’-TAGTCAGCCGTGGCAGCAGT-3’ |
| *CsUGT72F1* | GH6188.1 | F5’-GGCAAGAAGCTAATAGGGTCGTT-3’  R5’-TTGTATCATTCGGAAGTGGTGGG-3’ |
| *CsUGT73E1* | cam_GLEAN_10007306  tie_GLEAN_10002511 | F5’-ATGCCACCATAATCACCACC-3’  R5’-GCTCTCgCAACCTTCGG-3’ |
| *CsUGT78E1* | cam_GLEAN_10010447 | F5’-GCACCATAACCACCCCACC-3’  R5’-TGTCACAAACACACCAACCGAT-3’ |
| *CsSCPL1* | Singletons 45342 | F5’-GCTCATCTTCCTCCTAGTGC-3’  R5’-GGATTCCCTTCCGACTCTA-3’ |
| *CsSCPL2* | Singletons 1199 | F5’-CTGGCGGTTGGGAGTTAT-3’  R5’-CCTTGGCAGTGAGGTTCTT-3’ |
| *CsSCPL3* | cv699874 | F5’-TGGCGGTGATTCTTATTC-3’  R5’-AGTGTTAGCCTGTGAGCAA-3’ |
| *CsMYB4-1* | cam_GLEAN_10020745 | F5’-ATCCACAACGATAAACCG-3’  R5’-CGACATAGTCAGCCCAGA-3’ |
| *CsMYB4-2* | cam_GLEAN_10022034 | F5’-TTCTTAGCAGGGACCAGA-3’  R5’-AGGCGTACTCAACATTC-3’ |
| *CsMYB4-3* | cam_GLEAN_10033998 | F5’-AGATGAGGAAGAGGGCAGAG-3’  R5’-CCATTCGAGAAACACCAC-3’ |
| *CsMYB4-4* | tie_GLEAN_10016351 | F5’-ATTGAACCAAACACTTCCTCG-3’  R5’-GACGGAATGGCAATGGAG-3’ |
| *CsMYB4-5* | CsMYB3 | F5’-ATAAGTCTCCCAAATCCACCTC-3’  R5’-ATGATGCCCCGAAGAGC-3’ |
| *CsMYB4-6* | tie_GLEAN_10017628 | F5’-GAATACAAAACAGCGAAGAGTGC-3’  R5’-CAACCCTAAGAAATCATAACCAGTG-3’ |
| *CsMYB5-1* | cam_GLEAN_10020350 | F5’-GCTGTCATAACTTTGAACTCCAC-3’  R5’-GAATCAAGAAAGGATGCCAA-3’ |
| *CsMYB5-2* | tie_GLEAN_10028252 | F5’-GAATACAAAACAGCGAAGAGTGC-3’  R5’-CAACCCTAAGAAATCATAACCAGTG-3’ |
| *CsMYB7-1* | cam_GLEAN_10034384 | F5’-CCACCATTGTGAACTTGC-3’  R5’-CACCATCATTGGGGATT-3’ |
| *CsWD40-1* | tie_GLEAN_10026686 | F5’-CGGCAGCTTCATAGAGG-3’  R5’-TGGGAGGGTAAGGGTGT-3’ |
| *CsbHLH1-1* | cam_GLEAN_10016607 | F5’-TGGGAGTTTCGGGTGTT-3’  R5’-TTCAATCGGTCCTGTAAGA-3’ |
| *CsbHLH2-1* | cam_GLEAN_10012690  tie_GLEAN_10001220 | F5’-CCTATTCAACAACGCCTCC-3’  R5’-AGCGGTCATTGCTGTCTTC-3’ |
| *CsbHLH2-2* | cam_GLEAN_10006335  tie_GLEAN_10005499 | F5’-CTCCTAGACTTTGGTGTTCC-3’  R5’-GCCCCTGATTCAGATGAT-3’ |
| *CsbHLH3-1* | tie_GLEAN_10042091 | F5’-ATCTGATGAGAATTTGGACG-3’  R5’-TGGAAGAACACTACTGCTGAC-3’ |
| *CsbHLH3-2* | cam_GLEAN_10018241  tie_GLEAN_10006869 | F5’-CTGCGAGAAAAGGAAAGG-3’  R5’-TCCAAAAGTCATGGAACTG-3’ |
| *CsbHLH3-3* | cam_GLEAN_10018117  tie_GLEAN_10029282 | F5’-AGCGAGTGAAGACGCTT-3’  R5’-TTGTCACCCTCACCATAGA-3’ |
| *CsbHLH24-1* | tie_GLEAN_10024436 | F5’-CTGTTGGAATGGGAATGAGGA-3’  R5’-CGTGGGGTAGGGAAAAGTGT-3’ |
| *CsbHLH24-2* | tie_GLEAN_10022821  cam_GLEAN_10020333 | F5’-TATGTGCCTACCTGGAGTCTTGC-3’  R5’-GGGTATTTGTCGGAGTTTCTTG-3’ |
| *CsbHLH24-3* | cam_GLEAN_10025059 | F5’-AGTGCTGAATACAAACGGAGGA-3’  R5’-TGGTGTTAGGAGATTTGGCTGA-3’ |
| *CsbHLH24-4* | tie_GLEAN_10042126 | F5’-CAGGCACAGGCAACTTCAGA-3’  R5’-GAACATGGCTTGATGGCAAA-3’ |
| *CsbHLH24-5* | cam_GLEAN_10023958  tie_GLEAN_10006711 | F5’-TCTCCCAGAAAACCCAAACC-3’  R5’-CCAGAGCTTCAAAACCCTCC-3’ |
